# One-, two- and three-dimensional interlocked polymers based on hybrid inorganic–organic rotaxanes†

**DOI:** 10.1039/d4cc03566f

**Published:** 2024-08-09

**Authors:** Selena J. Lockyer, George F. S. Whitehead, Grigore A. Timco, Eric J. L. McInnes, Richard E. P. Winpenny

**Affiliations:** a Department of Chemistry, The University of Manchester Oxford Road Manchester M13 9PL UK selena.lockyer@manchester.ac.uk richard.winpenny@manchester.ac.uk

## Abstract

We report three new polymers, based on mechanically interlocked inorganic–organic rotaxanes. They are made in very mild conditions and involve pyrimidine head groups binding to copper(ii) linking units. A two-dimensional 6,3 net and a three-dimensional 10,3b net are found depending on the solvent used in the reaction.

## Abstract 2

We report three new polymers, based on mechanically interlocked inorganic–organic rotaxanes.

## Graphical abstract

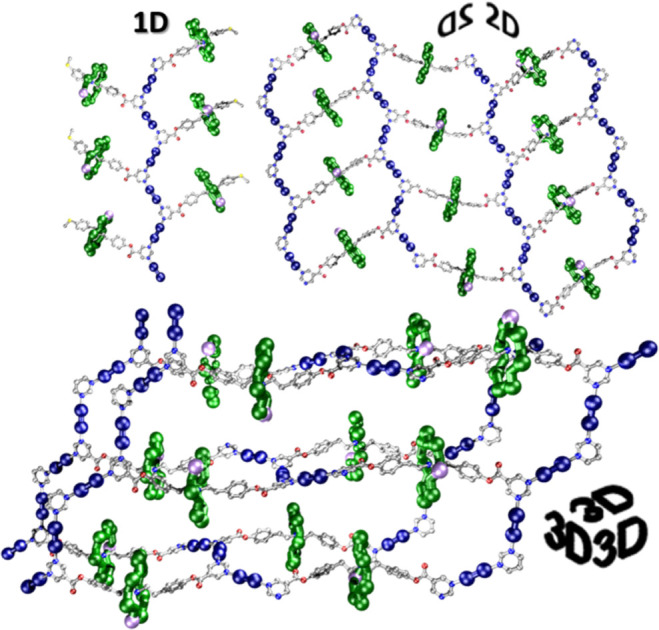

Metal–organic frameworks (MOFs) are one of the most active areas of chemistry with around 11 000 publications a year.^1^ They have been proposed for application in catalysis,^2^ separations^3^ and sensors.^4^ One class of MOF are mechanically interlocked molecules (MIMs); these can consist of one-,^5^ two-^6^ and three-dimensional^7–10^ coordination polymers, with non-covalently attached rings around the links that form the MOF. As the rings are held in place mechanically these MOFs are also known as rotaxane coordination polymers (RCP).

Most commonly, organic linkers are used between transition metal or lanthanide nodes.^11^ In almost all MIMS an organic molecule forms the macrocyclic component of a rotaxane.^12^ Sometimes a metal ion is incorporated to template the wheel to the axle, such as a Cu ion,^13,14^ but there is a recent report of MIMs based on {Al_8_} macrocycles.^15^ Many MIMs present challenges due to solubility, leading to use of polar solvents and/or solvothermal conditions. In our compounds the macrocyclic component is [Cr_7_NiF_8_(O_2_C^*t*^Bu)_16_]^−^1, and the presence of sixteen pivalates increases solubility in non-polar solvents, allowing structures to form in a range of organic solvents and under mild conditions, which is a current target for the development of MOFs.^1^ The {Cr_7_Ni} ring forms around an secondary ammonium, which can incorporate functionalised stoppers to directly bind to other molecules or can be further adapted once the ring has been formed. We have reported polymeric structures based on {Cr_7_Ni} rings, including 1D chains linked by Cu^II^ ions^16^ or {Fe_2_Co} triangles^17^ and a 10,3b net with {Fe_2_Co} nodes.^18^

Here, we report three new polymeric structures using two [2]rotaxanes containing 1 as the ring. The dimensionality of the resulting MOF is controlled by functionalisation of the thread with pyrimidine end groups, leading to a 1D-zig-zag chain, a 2D 6,3 net and a 3D 10,3b net. The nodes within these MIMs are all [Cu_2_(O_2_C^*t*^Bu)_4_], where the pivalates give high solubility.^19,20^

A {Cr_7_Ni} was grown around thread A (Fig. 1) to produce [2]rotaxane [(AH)1], 2; this can undergo a Steglich esterification to form the rotaxane [(CH)1], 3 (Fig. S1, ESI†). Compound 3 was dissolved in acetone, and one equivalent of [Cu_2_(O_2_C^*t*^Bu)_4_(HO_2_C^*t*^Bu)_2_] added, followed by addition of toluene. Slow evaporation gave crystals of the 1D polymer; {(3)[Cu_2_(O_2_C^*t*^Bu)_4_]}, 4. 4 crystallises in the *P*1̄ space group and forms a 1D chain (Fig. 1). The pyrimidine end group from the [2]rotaxane threads bridge between two {Cu_2_} paddlewheels. The pyrimidine-{Cu_2_} chains form the backbone of the structure, with the rotaxanes branching out in alternate directions. The distance between adjacent {Cr_7_Ni} rings (centre-to-centre) is 17.31(2) Å; that between opposing {Cr_7_Ni} rings is 27.09(3) Å.

**Fig. 1:**
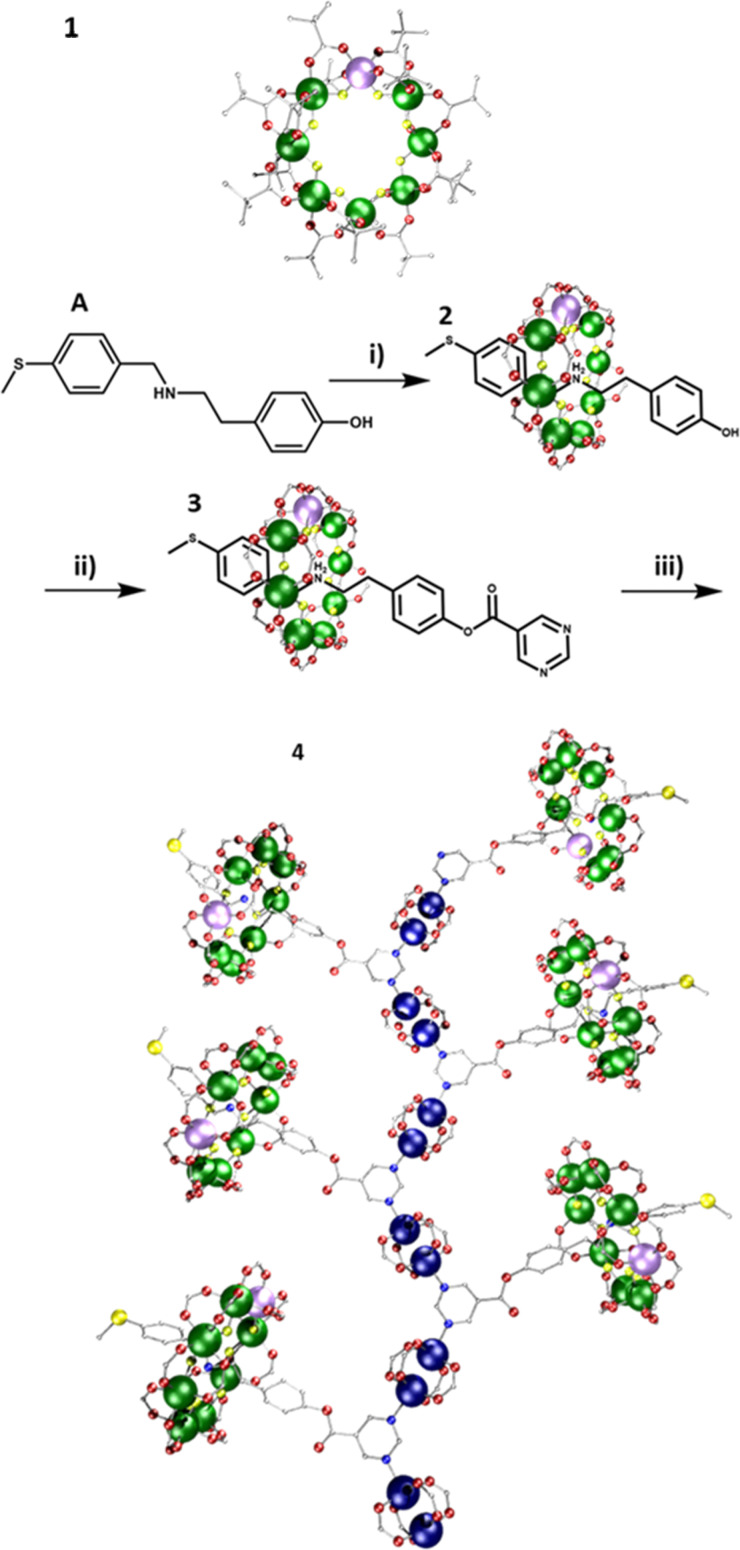
Scheme for synthesis of the 1D polymer 4. Reaction conditions: (i) pivalic acid, 2NiCO_3_·3Ni(OH)_2_·4H_2_O, CrF_3_·4H_2_O, 160 °C, 24 h; (ii) pyrimidine-5-carboxylic acid, DCC, DMAP, DCM, RT, 20 h; (iii) [Cu_2_(O_2_C^*t*^Bu)_4_(HO_2_C^*t*^Bu)_2_], acetone, toluene, RT; atom colours: Cr; green, Ni; violet, O; red, F; yellow, C; grey, S; dull yellow and N; blue. Hydrogens and ^*t*^Bu groups omitted for clarity.

A dual functionalised rotaxane was grown around thread B, to produce [(BH)1], 5 (Fig. 2). A double Steglich esterification gives the [2]rotaxane [(DH)1], 6 which includes a pyrimidine group at both ends of the thread (Fig. S2, ESI†). Compound 6 was dissolved in THF, and two equivalents of [Cu_2_(O_2_C^*t*^Bu)_4_(HO_2_C^*t*^Bu)_2_] added. MeCN was added, and slow evaporation gave crystals in 48 h of a 2D polymer {(6)[Cu_2_(O_2_C^*t*^Bu)_4_]_2_}, 7. 7 crystallises in the *P*2_1_/*n* monoclinic crystal system and forms 2D sheets. As in 4, the pyrimidine end groups coordinate to two {Cu_2_} units, and the pyrimidines and {Cu_2_} units produce a 1D-zig-zag chain. However, in 7 the chains are linked by [2]rotaxanes leading to a hexagonal 6,3 net (Fig. 2 and Fig. S14, ESI†). Hexagons are formed from two rotaxanes and four {Cu_2_} units. The 2D layers are stacked in an ABAB fashion. Disordered solvent molecules are found between the layers. The distance between equivalent {Cr_7_Ni} centres in neighbouring planes is 18.45(1) Å (Fig. S5, ESI†).

**Fig. 2:**
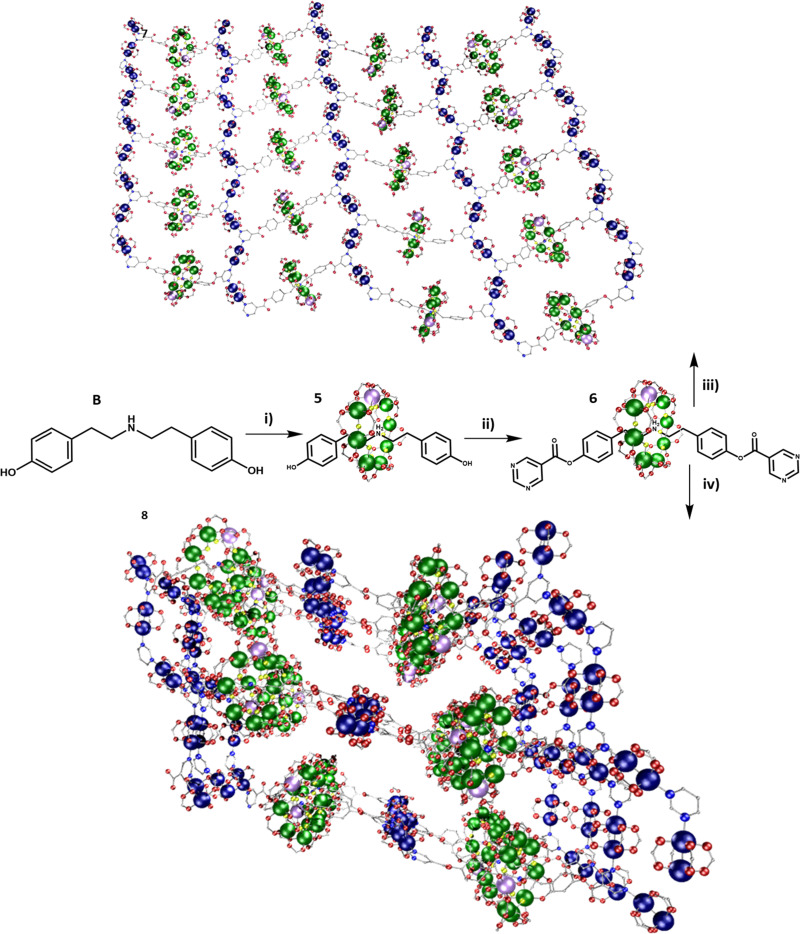
Scheme for synthesis of the 2D and 3D polymers; 7 and 8. Reaction conditions: (i) pivalic acid, 2NiCO_3_·3Ni(OH)_2_·4H_2_O, CrF_3_·4H_2_O, 160 °C, 24 h; (ii) pyrimidine-5-carboxylic acid, DCC, DMAP, DCM, RT, 20 h; (iii) [Cu(O_2_C^*t*^Bu)_4_(HO_2_C^*t*^Bu)_2_], thf, acetonitrile, RT; (iv) [Cu(O_2_C^*t*^Bu)_4_(HO_2_C^*t*^Bu)_2_], acetone, RT. Colours as Fig. 1.

The same reaction that formed 7 was carried out using acetone to dissolve the rotaxane and {Cu_2_}. In this case small crystals formed within a few seconds; slow evaporation over 24 h allowed further crystal growth of the 3D polymer; {(6)_2_[Cu_2_(O_2_C^*t*^Bu)_4_]_3_}, 8 (Fig. 2). 8 crystallises in the *P*2_1_/*n* monoclinic crystal system and forms a 3D MOF (Fig. S10–S12, ESI†). As in 4 and 7 the pyrimidine groups bind to two {Cu_2_}, however in 8 there are two distinct rows of {Cu_2_} units which are perpendicular to one another. This leads to a grid-like pattern of the {Cu_2_} framework. In order to complete a loop from one end of a rotaxane to the other end, the chain traverses an adjacent layer (Fig. S15, ESI†). Each circuit is made from four complete rotaxanes, six {Cu_2_} units and two pyrimidines from neighbouring rotaxanes, giving a 10,3b net. The ratio of {Cu_2_}:rotaxane did not affect whether 7 or 8 is formed, this is only controlled by the solvent. Compound 8 can be dissolved in THF (with addition of MeCN) to give 7. Compound 7 does not dissolve in acetone to give 8. This suggest that the formation of 8 is a result of the limited solubility that the {Cu_2_} and rotaxanes have in acetone when they initially bind. However, in THF the {Cu_2_} and rotaxane combination is much more soluble and allows time to rearrange to form the better packed structure of 7. While the three structures 4, 7 and 8 are 1-, 2- and 3-D respectively, the distances between the building blocks are consistent (Fig. 3 and Fig. S16, ESI,†Table 1). Firstly, the contacts from the {Cr_7_Ni} centroid to the nearest Cu(ii) have two distinct distances, one between 11.7–12.7 Å and one between 14.1–14.4 Å. Secondly, the {Cr_7_Ni}⋯{Cr_7_Ni} distance *via* {Cu_2_} is around 27 Å in all cases. The shortest {Cr_7_Ni}⋯{Cr_7_Ni} distance is through space in all three compounds, and is 16.6–17.3 Å. The inter-layer contacts between adjacent {Cr_7_Ni} rings in 4 is 17 Å, for 7 there are two distinct distances between layers, of 16 and 19 Å. Compound 8 does not contain individual layers. These contacts are short enough no spin-echo is seen in EPR experiments (see below).

**Fig. 3:**
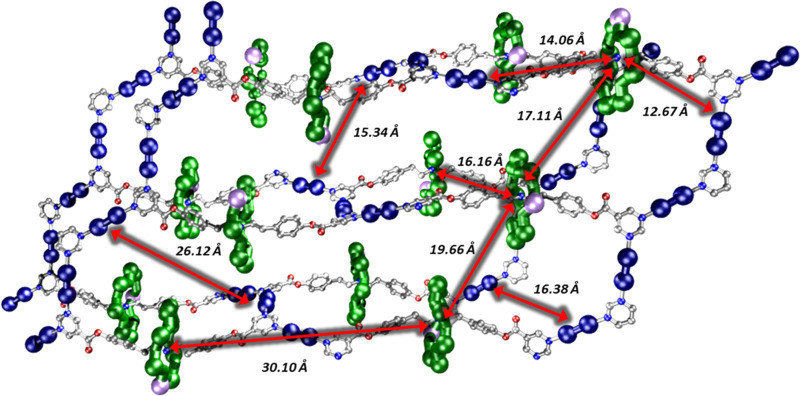
Section for the structure of 8, showing intra; ring–ring (center-centre) distances, ring-copper (centre to nearest contact) and copper paddle wheel-copper paddle wheel (nearest contacts).

**Table 1:** Contacts between building blocks in 4, 7 and 8

Compound	Contacts/Å		
	Ring centroid⋯Cu	Compound	Ring centroid⋯Cu
4	11.73, 14.37	4	11.73, 14.37
7	12.60, 14.16	7	12.60, 14.16
8	12.67, 14.06	8	12.67, 14.06

To study magnetic interactions between the building blocks, continuous wave (c.w.) EPR spectroscopy was performed at Q-band (*ca.* 34 GHz) on powder samples (Fig. S18, ESI†). At 5 K all three show only the well-defined spin *S* = 1/2 ground state of {Cr_7_Ni}.^16^ Strong anti-ferromagnetic coupling between the Cu^II^ ions within each {Cu_2_} renders this unit diamagnetic at 5 K. Above 10 K the resonance due to the *S* = 1/2 ground state of {Cr_7_Ni} decreases in intensity and a broad feature centred at *g* = 2.0 due to {Cr_7_Ni} excited states grows in. At 150 K the spectra are dominated by this broad feature, and a signal due to the *S* = 1 excited state of the {Cu_2_}^17^ is visible which grows in intensity on further warming. For 8 hyperfine coupling to the two *I* = 3/2 nuclear spins of the {Cu_2_} is clearly resolved on the Δ*m*_S_ = 2 transition at *ca.* 500 mT. The spectra for 4, 7 and 8 are almost identical, and are the sum of the spectra of the {Cr_7_Ni} and {Cu_2_} building blocks. No echo was observed in pulse EPR experiments on 4, 7 and 8 as powder samples at 3K. The EPR results indicate that although the interaction energies are too weak to be measured by c.w. EPR, they are still influencing the dynamic behaviour of the electron spins.

This work was supported by an EPSRC Established Career Fellowship (EP/R011079/1) to R.E.P.W. who also thanks the European Research Council for an Advanced Grant (ERC-2017-ADG-786734). We thank Diamond Light Source for access to synchrotron X-ray facilities. We also thank the EPSRC EPR NRF (EP/W014521/1, EP/X034623) and the Leverhulme trust for Grant RPG-2023-020.

## Data availability

The supporting data, including full experimental details, X-ray crystallography, CW EPR and EPR simulations have been included as part of the ESI.† Crystallographic data for 4, 7 and 8 have been deposited at the CCDC deposition service, under deposition numbers 2367045, 2367046 and 2367047, respectively.

## Conflicts of interest

The authors declare no conflict of interest.

## Supplementary Material

CC-060-D4CC03566F-s001

CC-060-D4CC03566F-s002
