# Supplementary material for: One-, two- and three-dimensional interlocked polymers based on hybrid inorganic–organic rotaxanes
Source: Chem Commun (Camb). 2024 Aug 9;60(74):10124–7. doi: 10.1039/d4cc03566f (PMC11348830; doi:10.1039/d4cc03566f)
Supplement: CC-060-D4CC03566F-s001 [file CC-060-D4CC03566F-s001.pdf]

Supplementary Information (SI) for ChemComm.
This journal is © The Royal Society of Chemistry 2024

Supporting Information

One-, Two- and Three-Dimensional Interlocked Polymers based on Hybrid Inorganic-Organic Rotaxanes

Selena J. Lockyer, G. F. S. Whitehead, Grigore A. Timco, Eric J. l. McInnes, Richard, E. P. Winpenny.

School of chemistry

School of Chemistry and Photon Science Institute, The University of Manchester, Oxford Road, Manchester M13 9PL, U.K.

CONTENTS:

1. Experimental Section **S2**

1.1 Organic secondary amine thread synthesis S2

1.2 Heterometallic Ring Synthesis S5

1.3 Esterification of Rotaxanes S7

1.4 Synthesis of $\{Cr_7Ni\}$ ring based polymer complexes S9

2. Crystallography Section **S10**

2.1 Crystallography Table S10

2.2 Structures S11

3. Electron Paramagnetic Resonance Measurements **S23**

4. References **S26**

1) Experimental Section

General remarks: All starting reagents and materials used were sourced from Sigma-Aldrich and/or Alfa. Unless stated otherwise, all reagents and solvents were used without further purification. The syntheses of the hybrid organic-inorganic rotaxanes were carried out in Erlenmeyer Teflon® FEP flasks supplied by Fisher. Column chromatography was performed using either 40-63 µm silica from Sigma-Aldrich or a Grace Reverelis ® X2 Autocolumn with Grace Reverelis ® NP cartridges. Chemical shifts are reported in parts per million (ppm) from low to high frequency and referenced to the residual solvent resonance. ESI mass spectrometry and microanalysis were carried out by the services at The University of Manchester.

Synthetic methods:

$[Cu_2(O_2{}^tBu)_4(HO_2{}^tBu)_2$ was prepared by previously reported methods.[S1]

1.1 Organic secondary amine thread synthesis [RNHR']:

1.1.1 Synthesis of **A** $[HO(C_6H4)CH_2CH_2NHCH_2(C_6H_4)SCH_3]$:

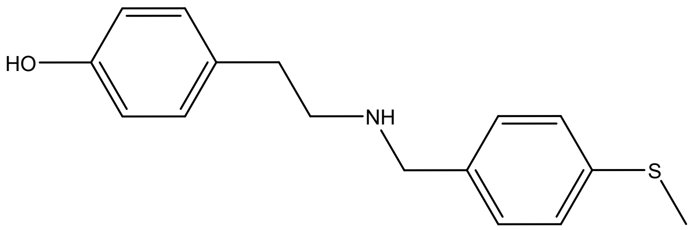

Under a flow of N_2, tyramine (2g, 14.6 mmol) was added to 2-Neck RBF, then dissolved in MeOH (100 mL). 4-(methylthio)benzaldehyde (2.23 g, 14.6 mmol) was then added and the solution refluxed for 4 hours at 80 °C. The solution was then left to cool to RT, then $NaBH_4$ (2.21g, 58 mmol) was added and left to stir for 12 hours at RT. The reaction was then quenched with H_2O (50 mL) while stirred for 1 hour. The product was extracted with DCM (3 x 30 mL), the organic phases combined and dried with $MgSO_4$. The solvents were then removed by reduced pressure to yield the powder product. Yield 3.43 g (86%). ESI MS m/z (relative intensity) 274 $[M+H]^+$, 296 $[M+Na]^+$. ^1H NMR (400 MHz, DMSO-d_6, 300 K): δ 9.14 (1H, Ph-OH), 7.24 (2H, Ar), 7.19 (2H, Ar), 6.96 (2H, Ar), 6.65 (2H, Ar), 3.75 (2H, CH_2), 2.60 (4H, CH_2CH_2), 2.45 (3H, CH_3).

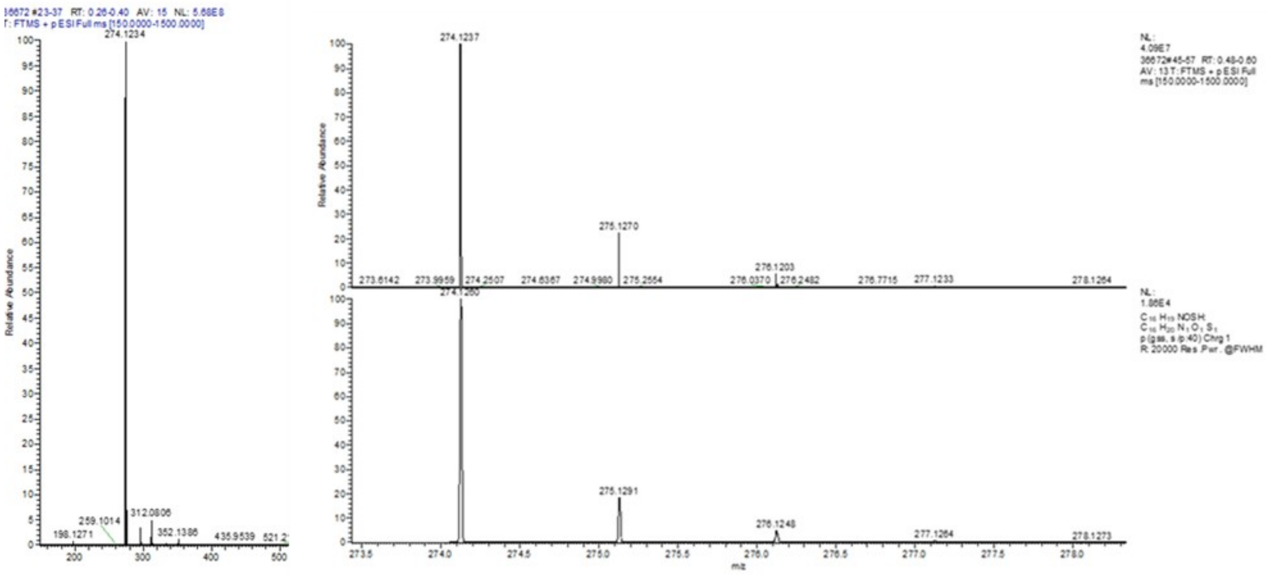

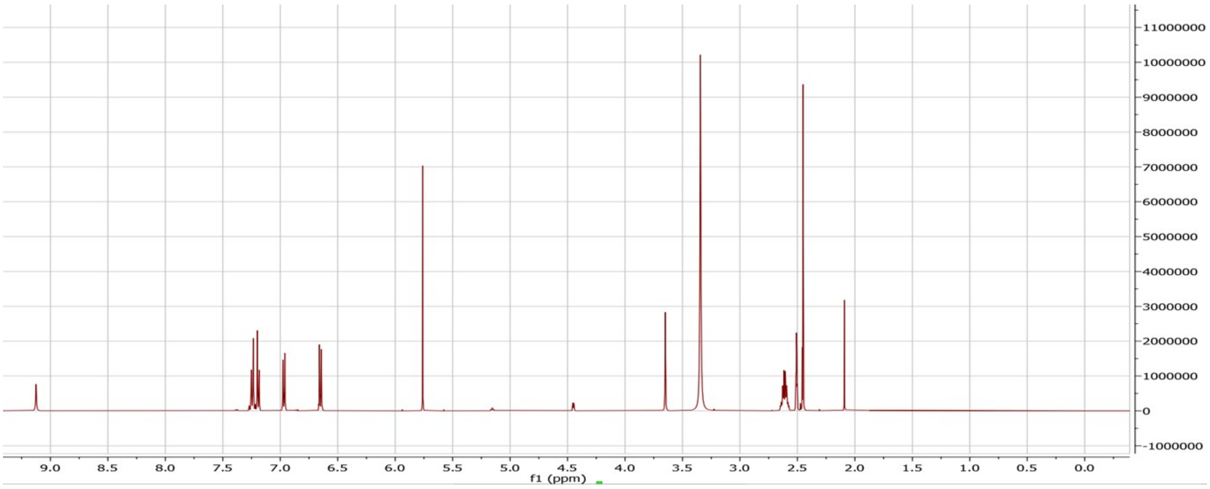

1.1.2 Synthesis of **B** [$HO(C_6H_4)CH_2CH_2NHCH_2CH_2(C_6H_4)OH$]:

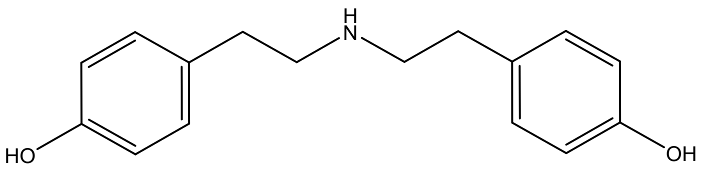

Under a flow of N_2, tyramine (11 g, 80 mmol) was added to a 2-Neck RBF under N2 flow, then dissolved in DMF (100 mL). Trimethylamine (10 mL) was then added and the mix heated to 70 °C. Next, 4-(2-bromethyl)phenol (4 g, 20 mmol) was added and the solution kept at 70 °C for 20 hours. After cooling to RT the product was extracted using EtOAc (2 x 100 mL) and H_2O

(100 mL), with washing from H_2O (2 x 100 mL). The organic layer was thgen dried with MgSO4, filtered and then the solvents were removed by reduced pressure, to yield the powder product. Yield: 3.21 g (62%). ESI MS m/z (relative intensity) 256 $[M-H]^-$. ^1H NMR (400 MHz, DMSO-d_6, 300 K): δ 9.15 (2H, Ph-OH), 6.94 (4H, Ar), 6.66 (4H, Ar), 2.67 (4H, CH_2), 2.55 (4H, CH_2).

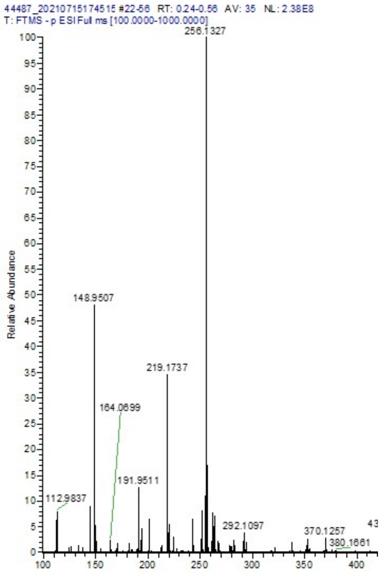

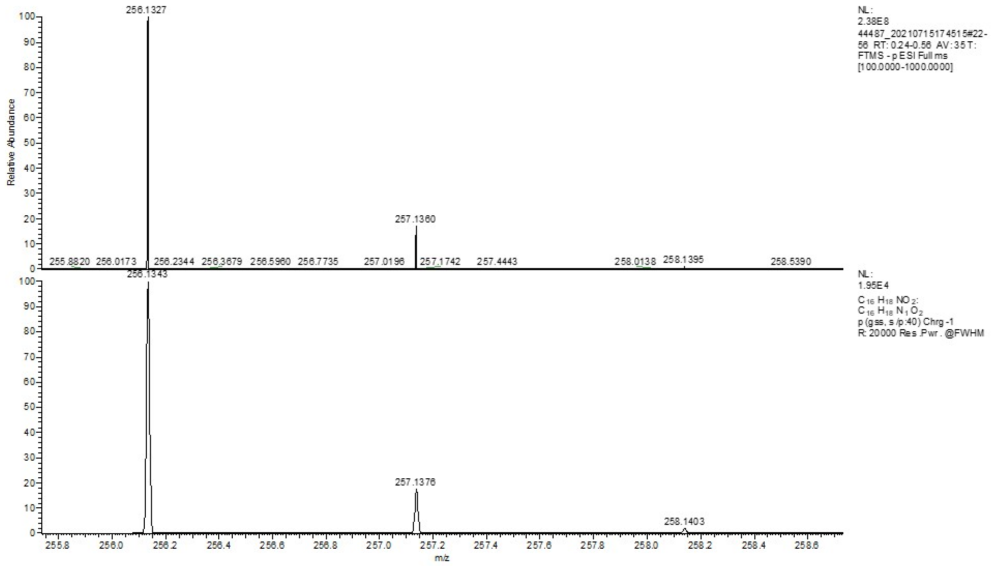

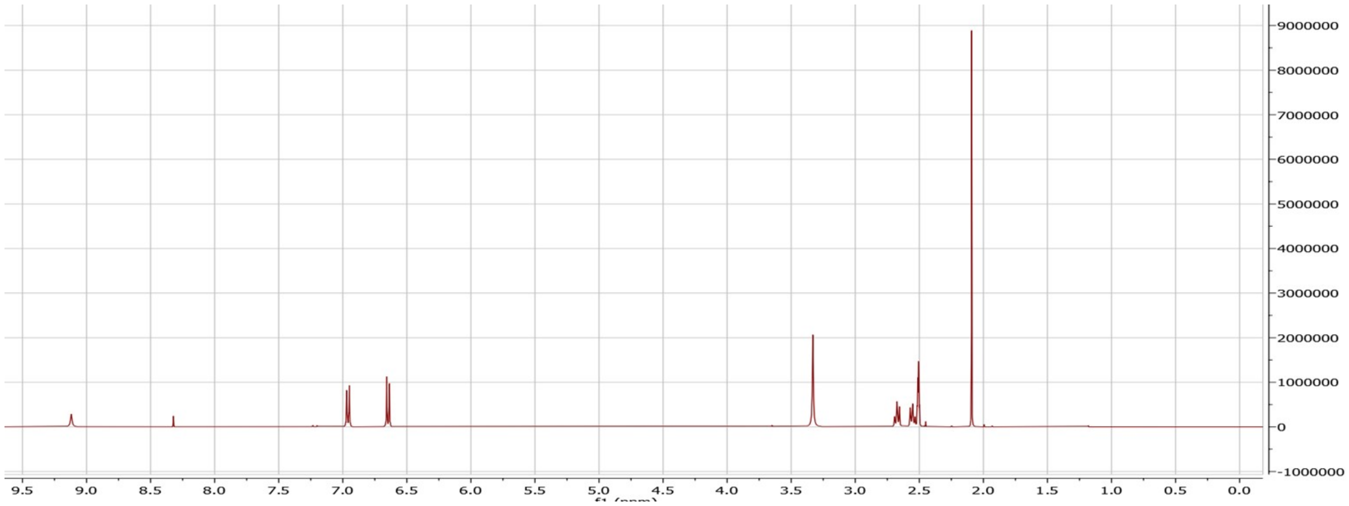

1.2 Heterometallic Ring Synthesis: (R,R'NH_2)[$Cr_7NiF_8(O_2C^tBu)_{16}$]:

1.2.1 Synthesis of **2**, [(**A**H)**1**]:

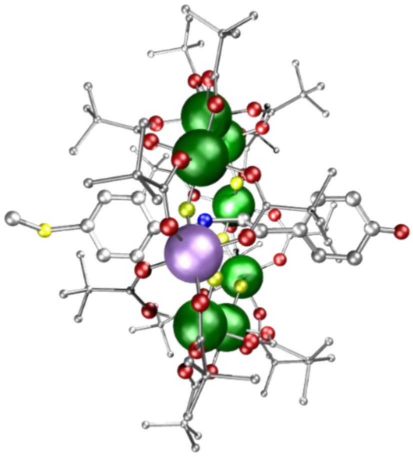

Pivalic ($^tBuCO_2H$) Acid (25 g, 245 mmol), $CrF_3.4H_2O$ (3 g, 16 mmol), $2NiCO_3.3Ni(OH)_2.4H_2O$ (0.35 g, 2.98 mmol) and **A** (1 g, 3.66 mmol) were added to a Teflon flask and heated to 160 °C for 24 hours. The mixture was left to cool to room temperature; acetonitrile (100 mL) was added, then stirred for 30 minutes. The mixture was filtered, with washing from acetonitrile (150 mL). Then extracted with DCM (150 mL), solvents were removed by reduced pressure to produce a dry green metallic powder. Yield: 1.56 g, 80%. ESI MS m/z (relative intensity) 2468 $[M+H]^+$, 2490 $[M+Na]^+$ 2506 $[M+K]^+$. Elemental analysis for $C_{96}H_{163}Cr_7F_8NNiO_{33}S$ (calculated)

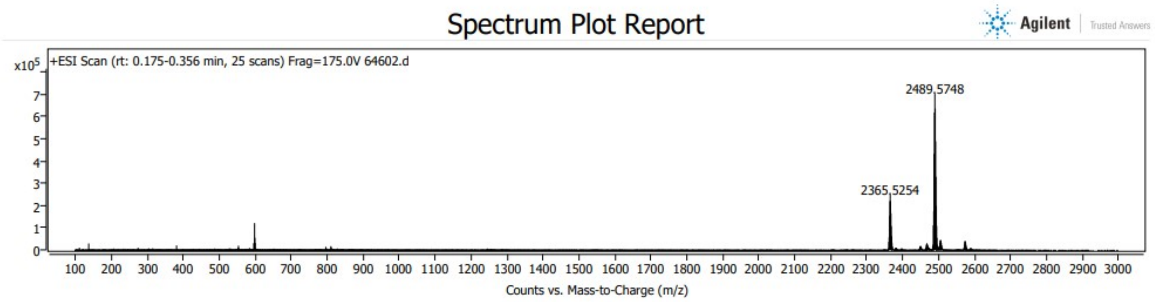

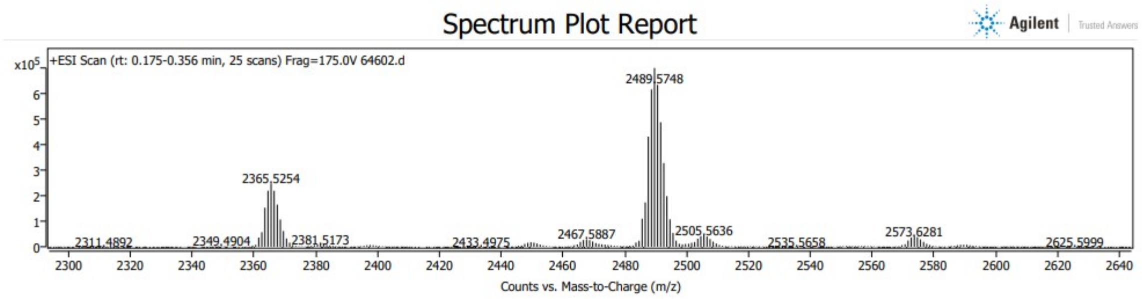

found: %, C: (46.76) 47.27, H: (6.66) 7.03, N: (0.57) 0.70, S: (1.30) 1.22, Cr: (14.76) 12.63, Ni: (2.38) 3.25.

1.2.2 Synthesis of **5**, [(**B**H)**1**]:

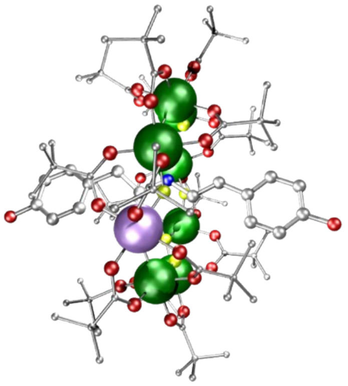

Pivalic ($^tBuCO_2H$) Acid (25 g, 245 mmol), $CrF_3.4H_2O$ (3 g, 16 mmol), $2NiCO_3.3Ni(OH)_2.4H_2O$ (0.35 g, 2.98 mmol) and **B** (1 g, 3.66 mmol) were added to a Teflon flask and heated to 160 °C for 24 hours. The mixture was left to cool to room temperature, acetonitrile (100 mL) was added, then stirred for 30 minutes. The mixture was filtered, with washing from acetonitrile (150 mL). Then extracted with DCM (150 mL), solvents were removed by reduced pressure to produce a dry green metallic powder. Yield: 1.25 g, 64%. ESI MS m/z (relative intensity) 2452 $[M+H]^+$, 2474 $[M+Na]^+$ 2490 $[M+K]^+$. Elemental analysis for $C_{96}H_{164}Cr_7F_8NNiO_{34}$ (calculated) found: %, C: (47.04) 46.70, H: (6.74) 6.88, N: (0.57) 0.53, Cr: (14.85) 14.18, Ni: (2.39) 2.06.

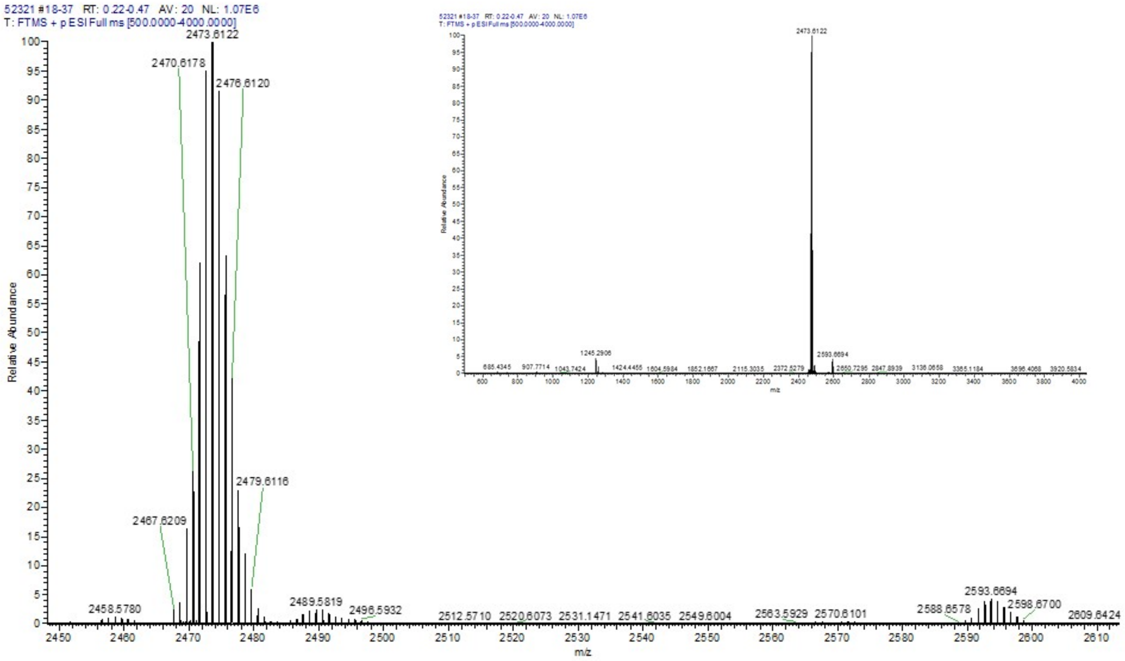

1.3 Esterification of Rotaxanes: (R,R'NH_2)[$Cr_7NiF_8(O_2C^tBu)_{16}$]:

1.3.1 Synthesis of **3**, [(**C**H)**1**]:

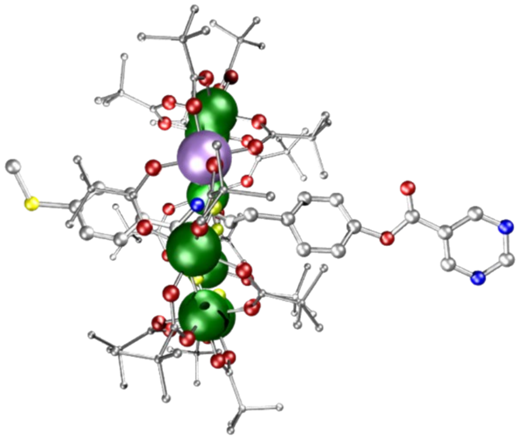

Pyrimidine-5-carboxylic Acid (0.136 g, 1.09 mmol) was dissolved in DCM (100 mL) under a flow of N_2. Then 4-dimethylaminopyridine (0.134 g 1.09 mmol) and N,N'-dicyclohexycarbodiimide (0.226 g, 1.09 mmol) was added. After stirring at RT for 30 minutes **2** (0.9 g, 0.365 mmol) was added and the solution was stirred at RT for 24 hours. The solvent was removed and the resulting green powder washed with acetonitrile. The product was purified by column chromatography, DCM:EtOAc (5:1) gave the second fraction as the product. The solvent was removed to produce a green powder. Yield: 0.78 g, 83%. ESI MS m/z (relative intensity) 2573 $[M+H]^+$, 2595 $[M+Na]^+$, 2611 $[M+K]^+$. Elemental analysis for $C_{101}H_{166}Cr_7F_8N_3NiO_{34}S$ (calculated) found: %, C: (47.14) 47.36, H: (6.50) 7.04, N: (1.63) 1.78, S:

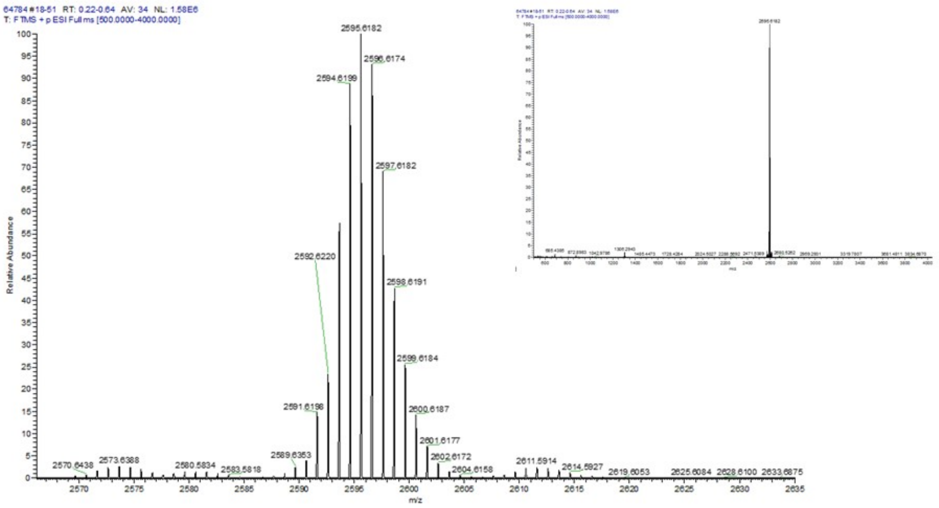

(1.25) 1.31, Cr: (14.15) 13.54, Ni: (2.28) 2.24.

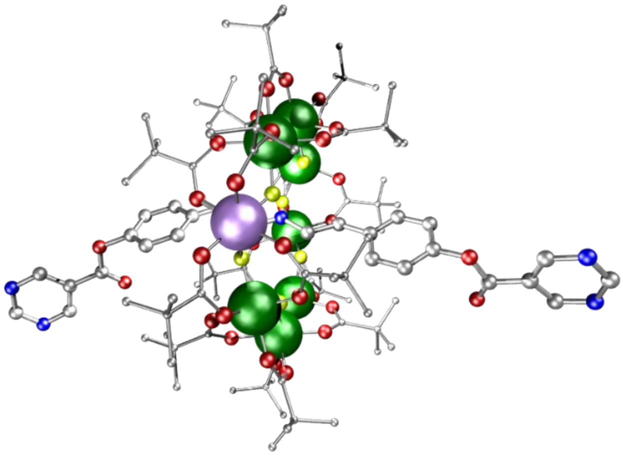

1.3.2 Synthesis of **6**, [(**D**H)**1**]]:

Pyrimidine-5-carboxylic Acid (0.304 g, 2.45 mmol) was dissolved in DCM (100 mL) under a flow of N_2. Then 4-dimethylaminopyridine (0.300 g 2.45 mmol) and N,N'-dicyclohexycarbodiimide (0.510 g, 2.45 mmol) was added. After stirring at RT for 30 minutes **5** (1.0 g, 0.408 mmol) was added and the solution was stirred at RT for 24 hours. The solvent was removed and the resulting green powder washed with acetonitrile. The product was purified by column chromatography, DCM:EtOAc (4:1) gave the third fraction as the product. The solvent was removed to produce a green powder. Yield: 0.62 g, 60%. ESI MS m/z (relative intensity) 2663 $[M+Na]^+$, 2680 $[M+K]^+$. Elemental analysis for $C_{106}H_{168}Cr_7F_8N_5NiO_{36}$ (calculated) found: %, C: (47.81) 47.27, H: (6.36) 6.43, N: (2.63) 2.70, Cr: (13.67) 13.13, Ni: (2.20) 2.20.

1.4 Synthesis of $\{Cr_7Ni\}$ ring based polymer complexes:

1.4.1 Synthesis of **4**, 1D polymer, $\{(\mathbf{3})[Cu_2(O_2C^tBu)_4]\}$:

3 (0.052 g, 0.02 mmol) was dissolved in warm acetone (15 mL, 40 °C). Then $[Cu_2(O_2{}^tBu)_4(HO_2{}^tBu)_2]$ (0.03 g, 0.04 mmol) was added, and the solution was stirred for 10 minutes, then left to cool to RT. Toluene (5 mL) as then added and the solution left for slow evaporation. Crystals suitable for XRD were grow over 24 hours. Yield 0.021 g (32%) Elemental analysis for $C_{121}H_{202}Cr_7Cu_2F_8N_3NiO_{42}S$ (calculated) found: %C (46.81) 45.82; %H (6.56) 6.57;

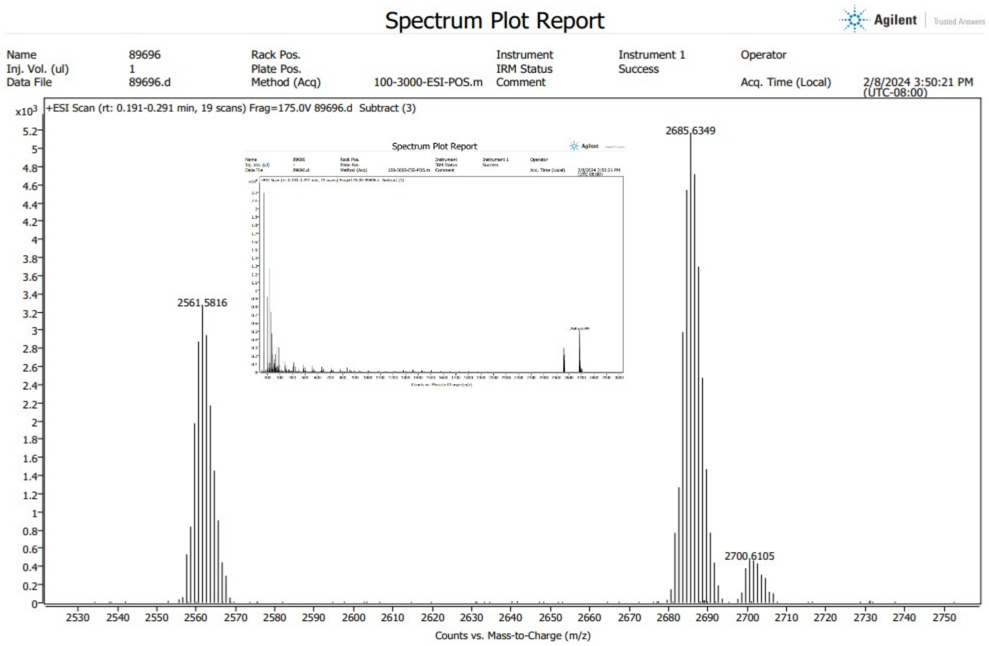

%N (1.89) 1.43; %S (1.03) 0.99; %Cr (11.72) 11.106; %Ni (1.89) 1.86; %Cu (4.09) 3.99.

1.4.2 Synthesis of **7**, 2D polymer, {(**6**)[$Cu_2(O_2C^tBu)_4$]$_2$}:

6 (0.08 g, 0.03 mmol) was dissolved in THF (10 mL) at RT. Then [$Cu_2(O_2{}^tBu)_4(HO_2{}^tBu)_2$] (0.045 g, 0.06 mmol) was added, and the solution was stirred for 10 minutes, before acetonitrile (5 mL) was added. Crystals suitable for XRD were grow via slow evaporation over 48 hours. Yield 0.04 g (44%) Elemental analysis for $C_{124}H_{204}Cr_7Cu_2F_8N_5NiO_{44}$ (calculated) found: %C (46.97) 47.25; %H (6.48) 6.67; %N (2.21) 2.34; %Cr (11.48) 10.71; %Ni (1.85) 1.83; %Cu (4.01) 3.96.

1.4.3 Synthesis of **8**, 3D polymer, {(**6**)$_2$[$Cu_2(O_2C^tBu)_4$]$_3$}:

4 (0.08 g, 0.03 mmol) was dissolved in acetone (15 mL) at RT. Then [$Cu_2(O_2{}^tBu)_4(HO_2{}^tBu)_2$] (0.045 g, 0.06 mmol) was added. Crystals suitable for XRD then began to form within a few minutes. Yield 0.05 g (56%) Elemental analysis for $C_{146}H_{240}Cr_7Cu_4F_8N_5NiO_{52}$ (calculated) found: %C (47.061) 46.21; %H (6.49) 6.67; %N (1.88) 1.97; %Cr (9.77) 9.25; %Ni (1.88) 1.59; %Cu (6.82) 6.61.

2) Crystallography Section:

X-ray diffraction data for compounds **4**, **7** and **8** were collected at Diamond Light Source beamline (I19) using Zr-L edge wavelength (λ = 0.6889Å) radiation at a temperature of 100 K. Data were collected using GDA. All data were reduced using CrysAlisPro v42.[S2,S3]

2.1 Crystallography Table:

Table S1. Crystallographic Data

Compound	**4**	**7**	**8**
Identification code	SJL637_7_8	SJL646c_5_c1	SJL646B_1_c1
Empirical formula	$C_{325}H_{504}Cr_{14}Cu_4F_{16}N_6Ni_2O_{86}S_2$	$C_{178}H_{302.48}Cr_7Cu_4F_8N_6NiO_{59.88}$	$C_{170.19}H_{290.65}Cr_7Cu_4F_8N_5NiO_{61.2}$
Formula weight	7339.01 g mol^{-1}	4313.63 g mol^{-1}	4215.08 g mol^{-1}
Temperature/K	100.00(10) K	100.00(10) K	100.00(10) K
Crystal system	triclinic	monoclinic	monoclinic
Space group	P-1	P2$_1$/*n*	P2$_1$/*n*
a/Å	16.5060(4)	35.2731(4)	26.9191(4)
b/Å	34.2036(11)	16.6464(2)	19.6641(3)
c/Å	35.1533(10)	37.7375(5)	43.9326(7)
α/°	80.444(2)	90	90
β/°	88.909(2)	94.682(1)	104.391(2)
γ/°	85.438(2)	90	90
Volume/Å^3	19508.6(10)	22084.4(5)	22441.9(6)
Z	2	4	4
ρ_{calc}g/cm^3	1.249	1.297	1.248
μ/mm^{-1}	0.709	0.804	0.790
F(000)	7736	9102	8882
Crystal size/mm^3	0.089 x 0.072 x 0.056	0.287 x 0.188 x 0.057	0.087 x 0.055 x 0.035
Radiation	synchrotron (λ = 0.6889)	synchrotron (λ = 0.6889)	synchrotron (λ = 0.6889)
2Θ range for data collection/°	2.908 to 40.296	3.168 to 42.052	3.142 to 50.3
Index ranges	-16 ≤ h ≤ 16, -34 ≤ k ≤ 34, -35 ≤ l ≤ 34	-36 ≤ h ≤ 36, -17 ≤ k ≤ 16, -39 ≤ l ≤ 39	-32 ≤ h ≤ 29, -23 ≤ k ≤ 23, -42 ≤ l ≤ 53
Reflections collected	136794	109027	91439
Independent reflections	40744 [R_{int} = 0.0929, R_{sigma} = 0.0880]	26087 [R_{int} = 0.0772, R_{sigma} = 0.0616]	32627 [R_{int} = 0.0773, R_{sigma} = 0.0903]
Data/restraints/parameters	40744/20612/3828	26087/12643/2675	32627/12415/2833
Goodness-of-fit on F^2	1.036	1.026	1.086
Final R indexes [I>=2σ (I)]	R_1 = 0.1014, wR_2 = 0.2826	R_1 = 0.0781, wR_2 = 0.2109	R_1 = 0.0699, wR_2 = 0.1952
Final R indexes [all data]	R_1 = 0.1366, wR_2 = 0.3093	R_1 = 0.1018, wR_2 = 0.2267	R_1 = 0.0937, wR_2 = 0.2111
Largest diff. peak/hole / e Å^{-3}	0.64/-0.43	0.71/-0.89	0.64/-0.72

2.2 Structures:

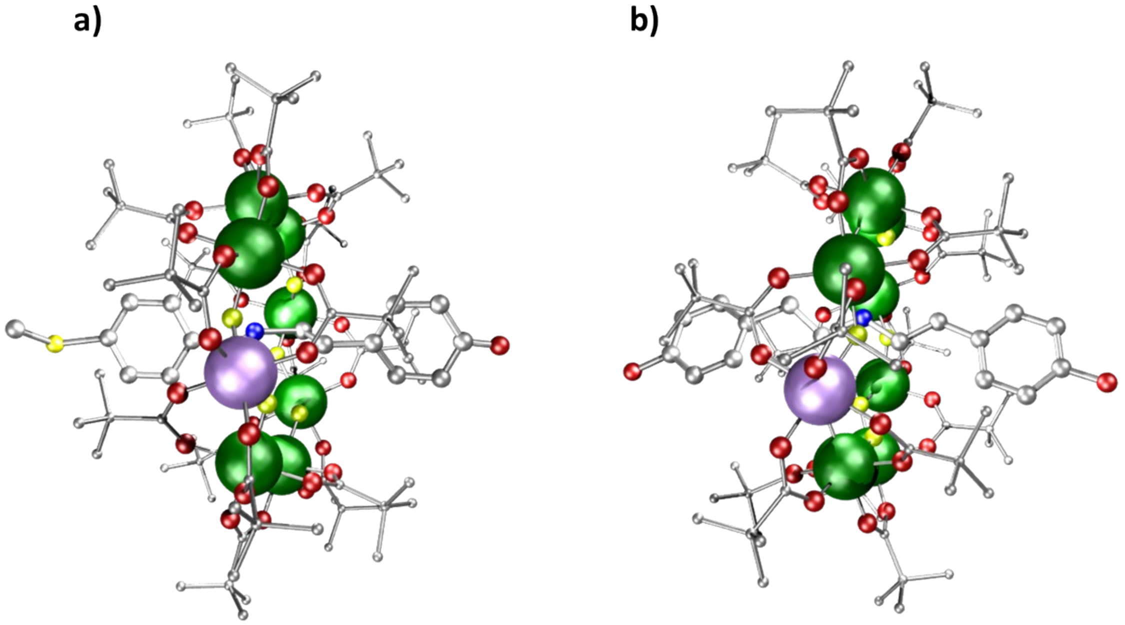

Figure S1. Structures for a) **2** and b) **5**. Atom colours: Cr; Green, Ni; Violet, O; Red, F; Yellow, C; Grey, N; Blue and S; Dull yellow. Hydrogens omitted for clarity.

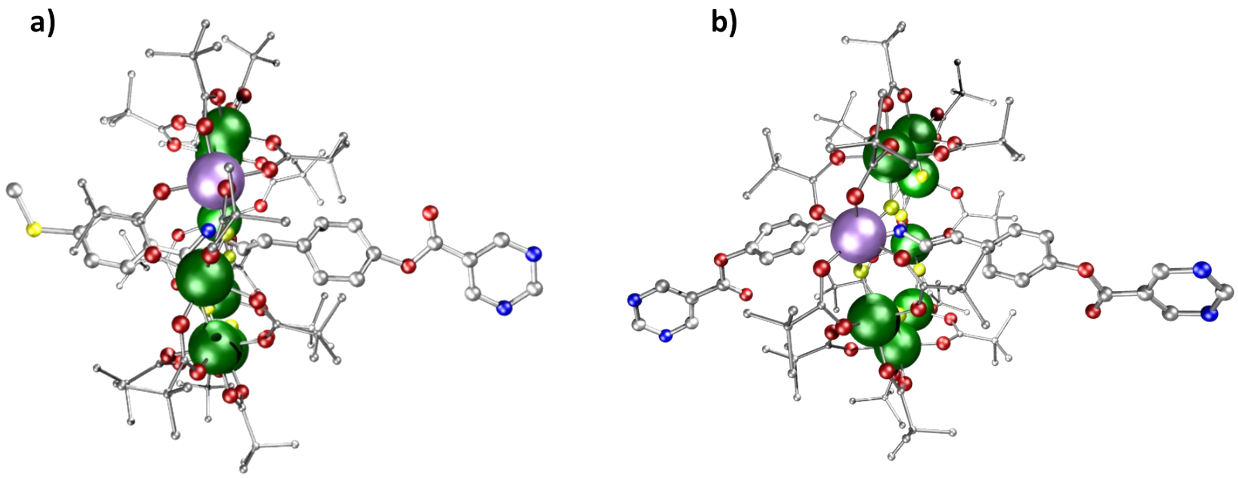

Figure S2. Structure for a) **3** and b) **6**. Atom colours: Cr; Green, Ni; Violet, O; Red, F; Yellow, C; Grey, N; Blue and S; Dull yellow. Hydrogens omitted for clarity.

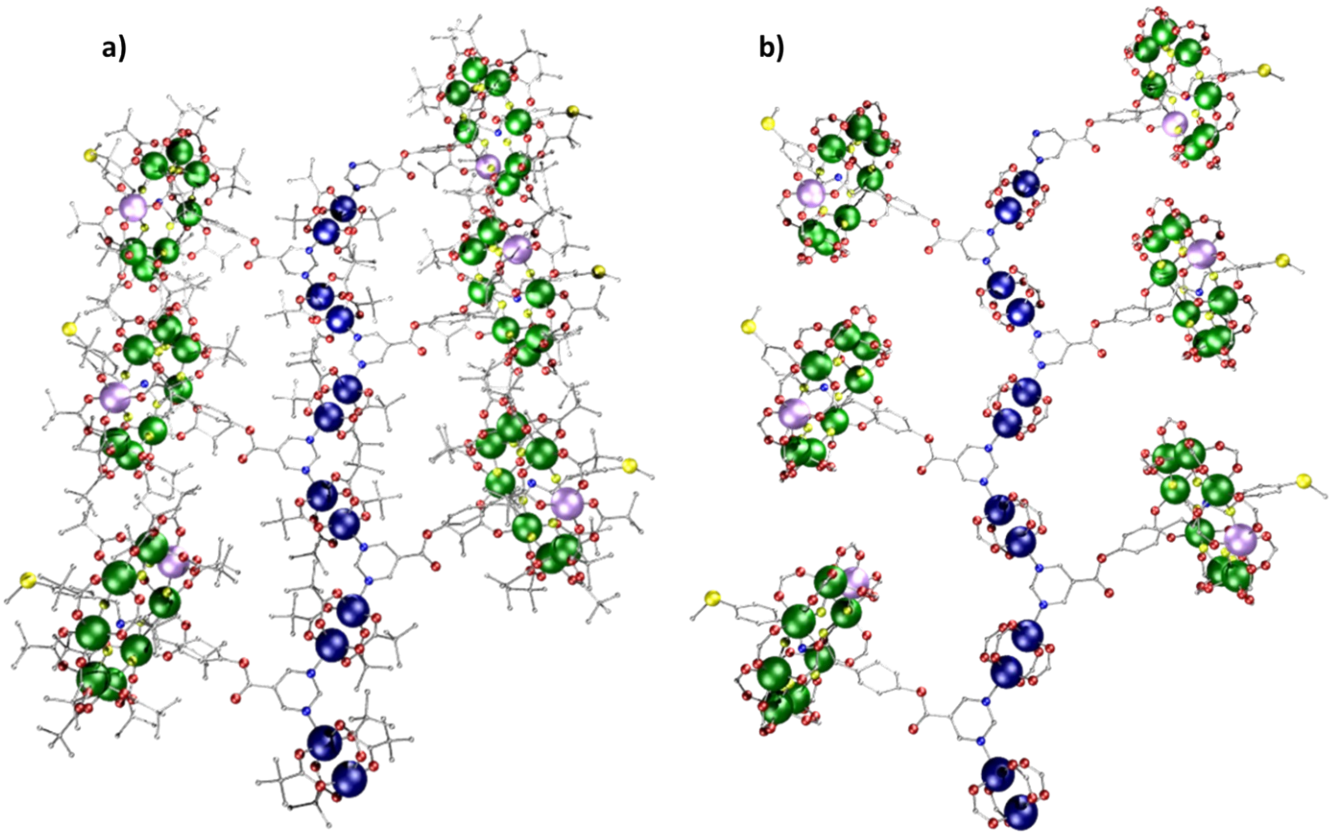

Figure S3. Crystal structure for **4**, a) with ^{t}Bu groups and b) without ^{t}Bu groups. Atom colours: Cr; Green, Ni; Violet, Cu; Navy blue, O; Red, F; Yellow, C; Grey, N; Blue and S; Dull yellow. Hydrogens omitted for clarity.

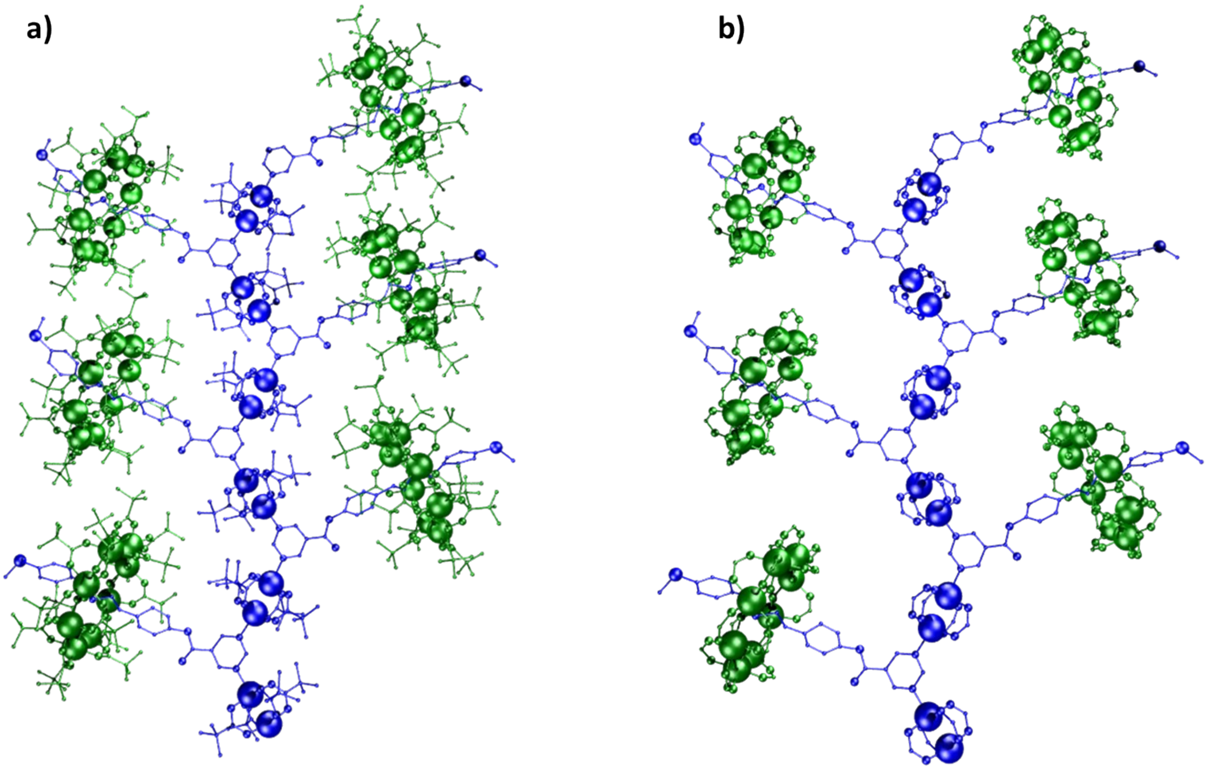

Figure S4. Crystal structure for **4**, a) with ^{t}Bu groups and b) without ^{t}Bu groups. Cu paddle wheel and thread of rotaxanes that make the framework in blue, the {Cr_7Ni} rings / wheel of the rotaxanes in green. Hydrogens omitted for clarity.

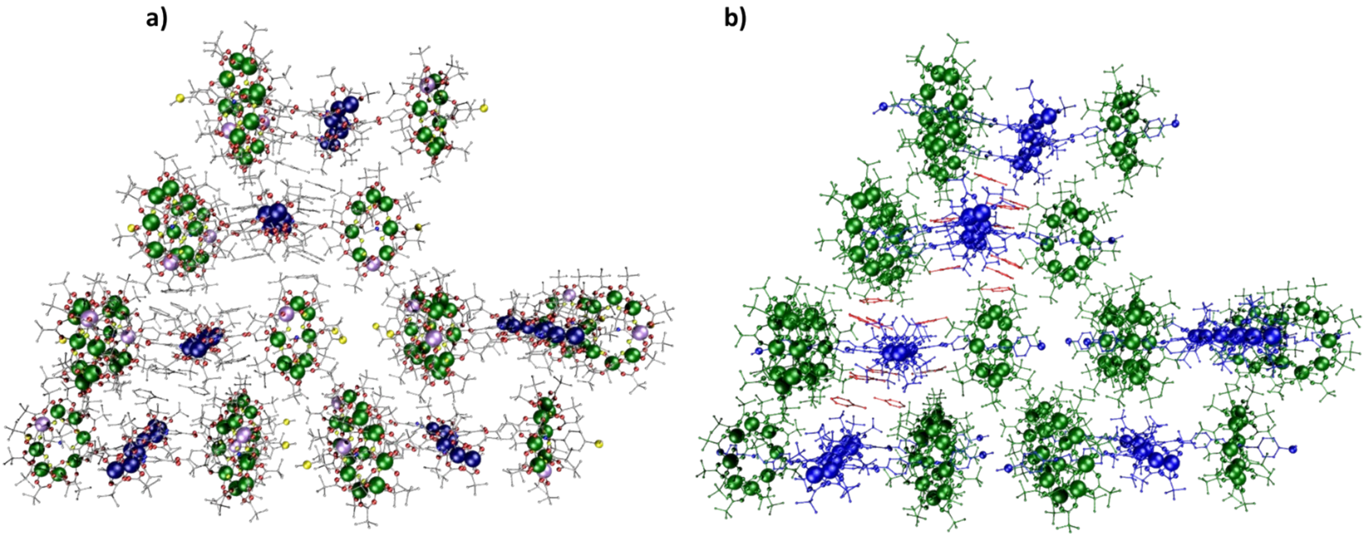

Figure S5. Crystal structure, showing the stacking of layers of the 1D chains for **4**, a) atom colours: Cr; Green, Ni; Violet, Cu; Navy blue, O; Red, F; Yellow, C; Grey, N; Blue and S; Dull yellow, b) Framework depicted in blue, {Cr_7Ni} rings in green and toluene molecules in red. Hydrogens omitted for clarity.

a) b)

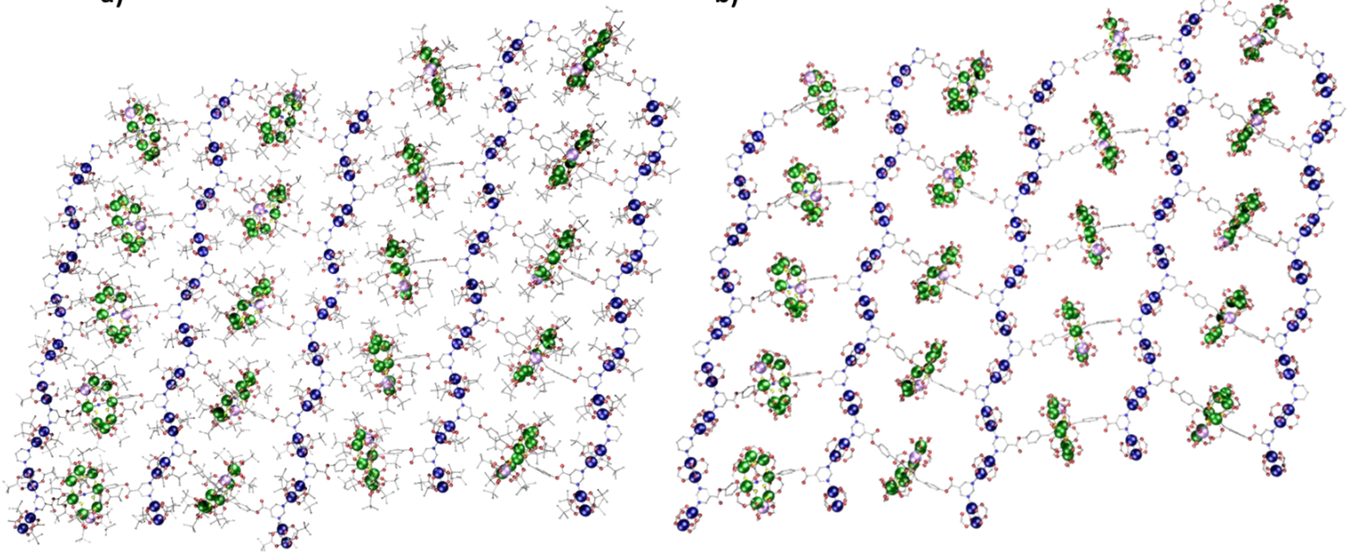

Figure S6. Crystal structure for **7** (one layer), a) with ^{t}Bu groups and b) without ^{t}Bu groups. Atom colours: Cr; Green, Ni; Violet, Cu; Navy blue, O; Red, F; Yellow, C; Grey and N; Blue. Hydrogens omitted for clarity.

a) b)

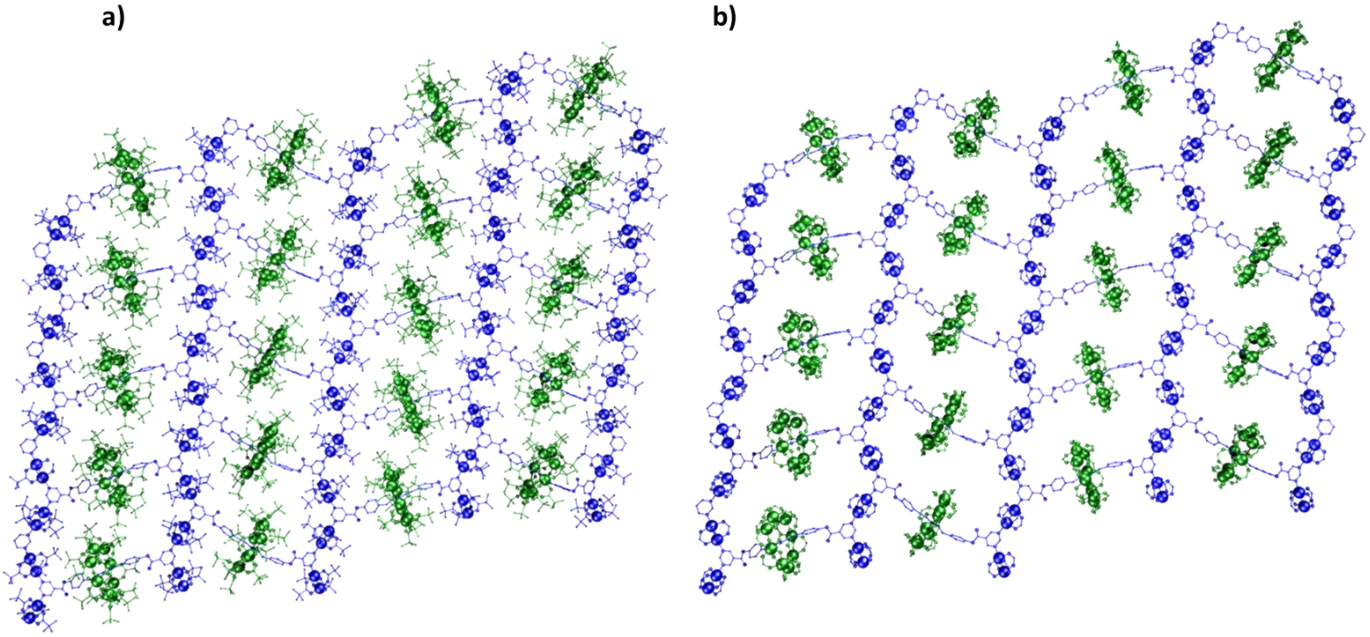

Figure S7. Crystal structure for **7** (one layer), a) with ^{t}Bu groups and b) without ^{t}Bu groups. Cu paddle wheel and thread of rotaxanes that make the framework in blue, the {Cr_7Ni} rings / wheel of the rotaxanes in green. Hydrogens omitted for clarity.

a)

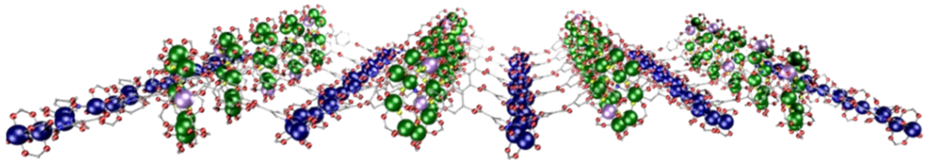

b)

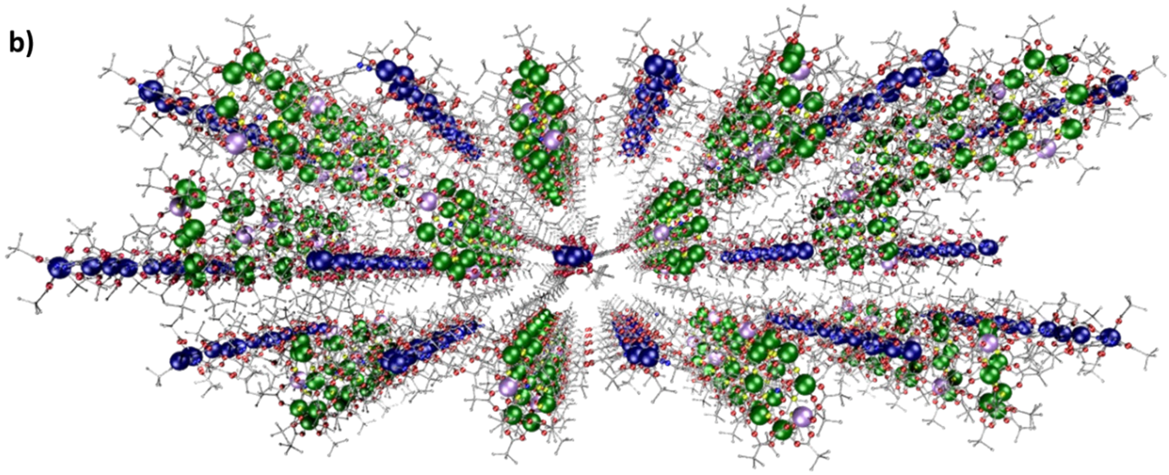

Figure S8. Crystal structure for **7** (end view), a) one layer (^{t}Bu groups and hydrogens omitted for clarity) and b) multiple layers showing ABAB stacking. Atom colours: Cr; Green, Ni; Violet, Cu; Navy blue, O; Red, F; Yellow, C; Grey and N; Blue, hydrogens omitted for clarity.

a)

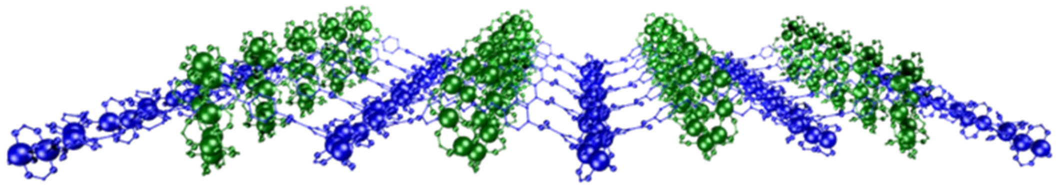

b)

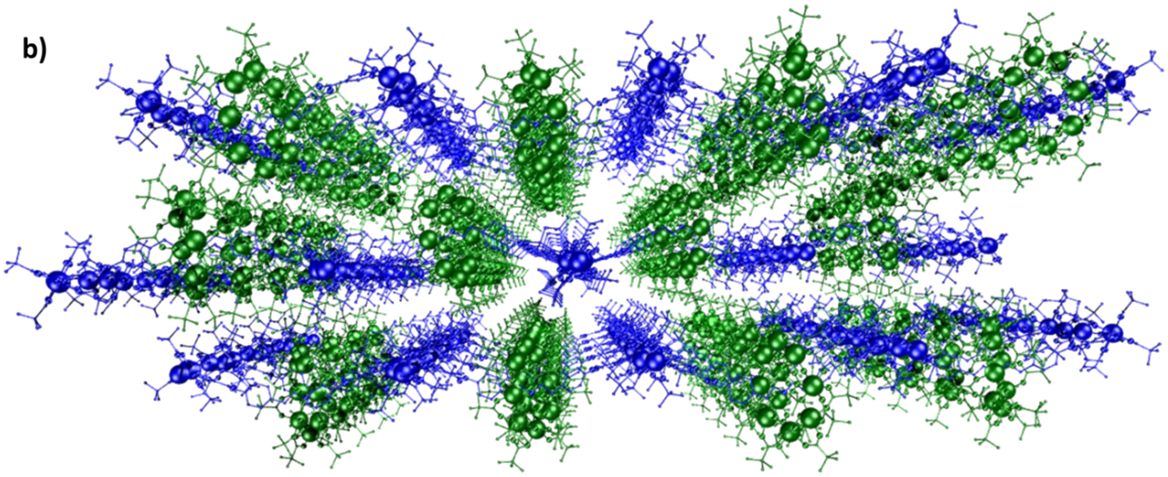

Figure S9. Crystal structure for **7** (end view), a) one layer (^{t}Bu groups and hydrogens omitted for clarity) and b) multiple layers showing ABAB stacking. Cu paddle wheel and thread of rotaxanes that make the framework in blue, the {Cr_7Ni} rings / wheel of the rotaxanes in green, hydrogens omitted for clarity.

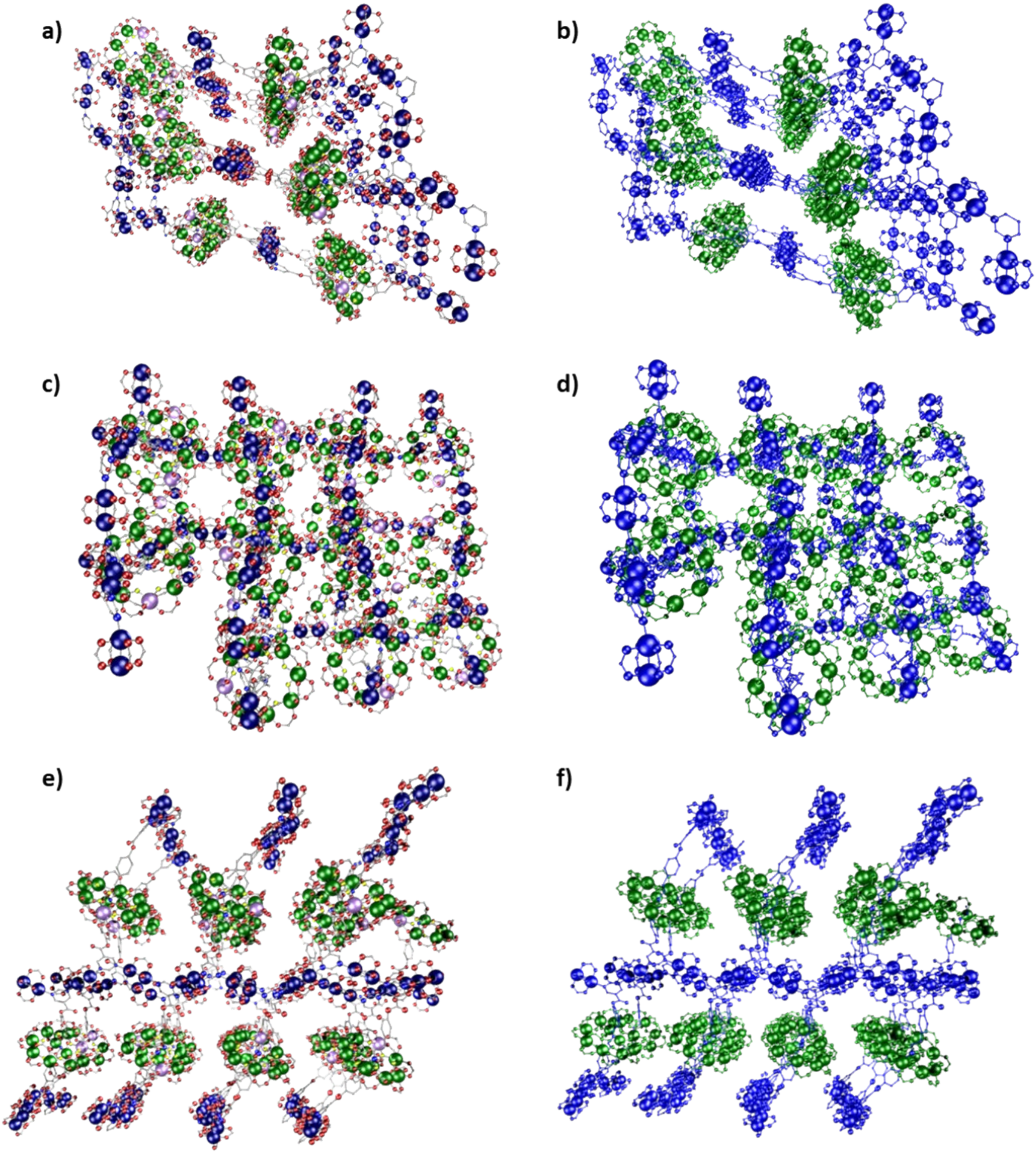

Figure S10. Crystal structure for **8.** Side, end and plane view for a) b), c) d), and e) f), respectively. Atom colours: Cr; Green, Ni; Violet, Cu; Navy blue, O; Red, F; Yellow, C; Grey and N; Blue, for a), c) and e). Cu paddle wheel and

thread of rotaxanes that make the framework in blue, the {Cr_7Ni} rings / wheel of the rotaxanes in green, for b), d) and f). ^{t}Bu groups and hydrogens omitted for clarity.

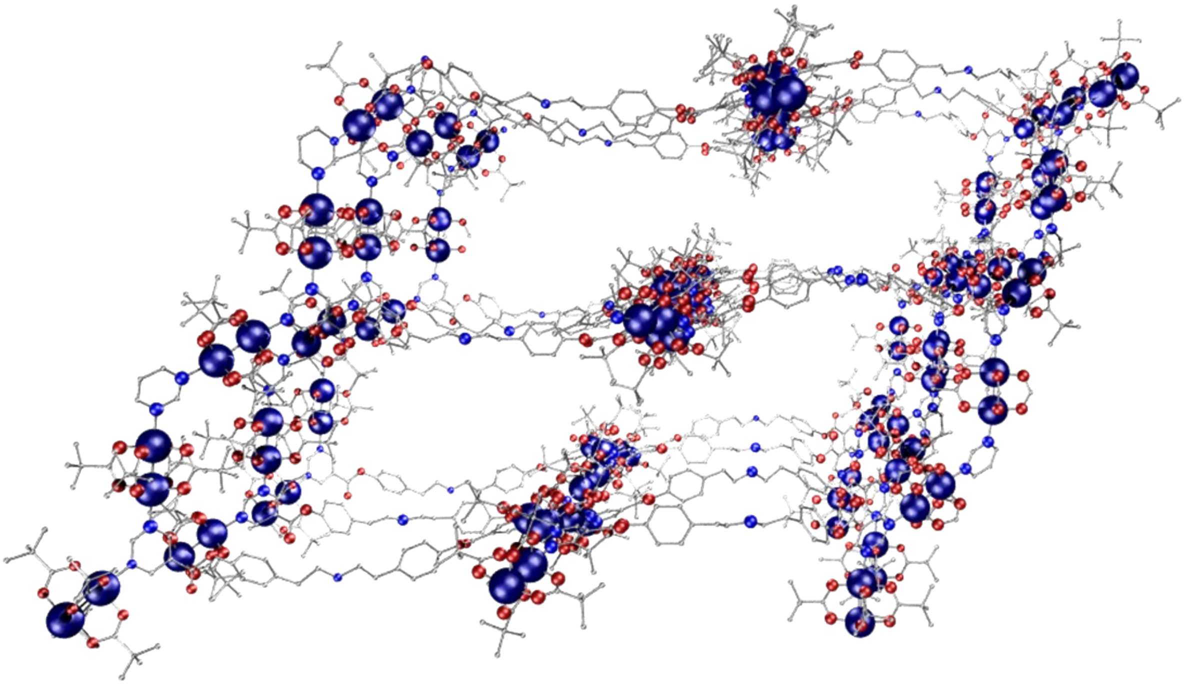

Figure S11. Crystal structure of just the framework consisting of the Cu paddle wheel and threads of the rotaxanes for **8**. Atom colours: Cu; Navy blue, O; Red, C; Grey and N; Blue. Hydrogens omitted for clarity.

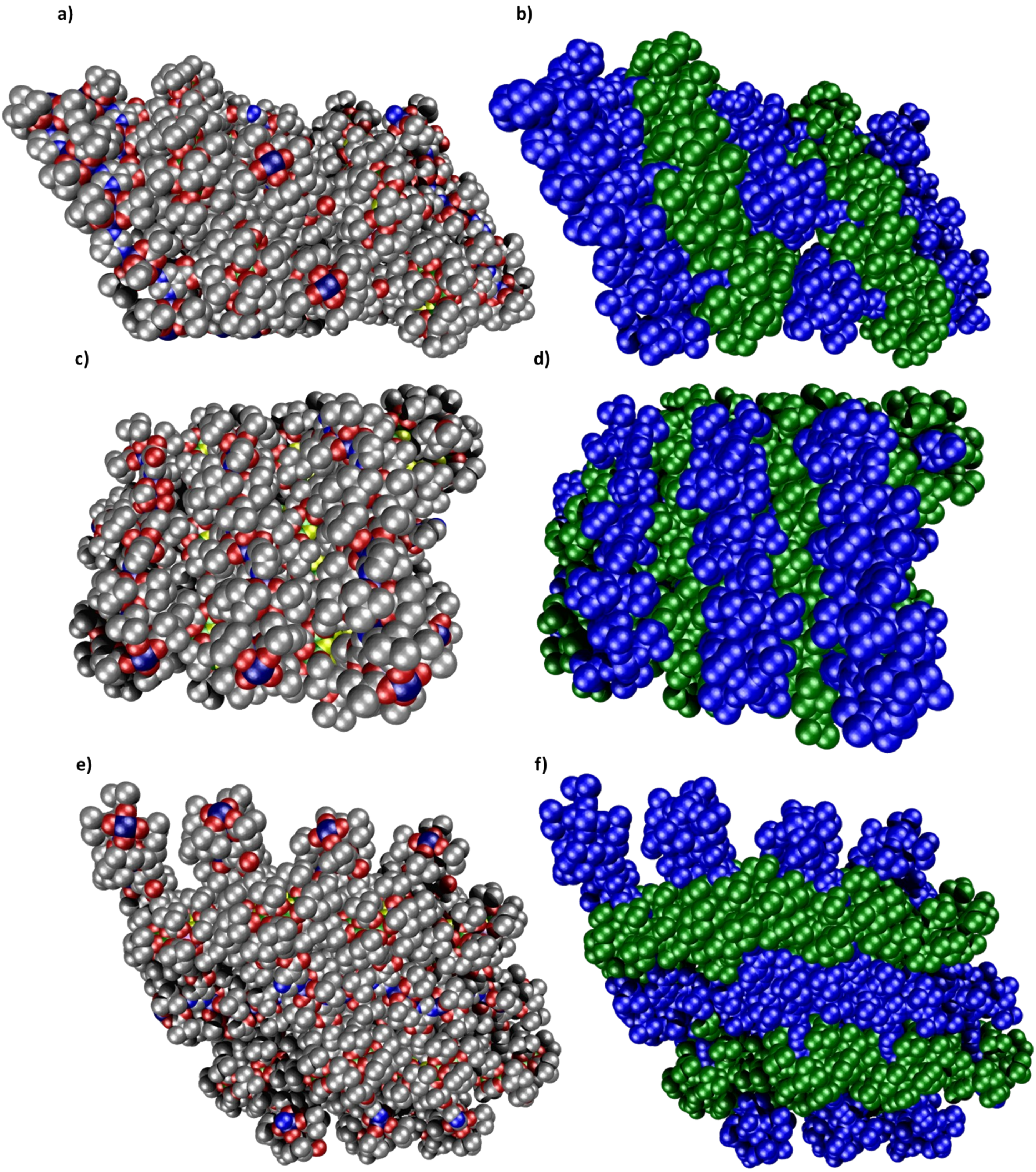

Figure S12. Space filling model of the crystal structure for **8**. Side, end and plane view for a) b), c) d), and e) f), respectively. Atom colours: Cr; Green, Ni; Violet, Cu; Navy blue, O; Red, F; Yellow, C; Grey and N; Blue, for a), c) and e), respectively. Cu paddle wheel and thread of rotaxanes that make the framework in blue, the {Cr_7Ni} rings / wheel of the rotaxanes in green, for b), d) and f), respectively. Hydrogens omitted for clarity.

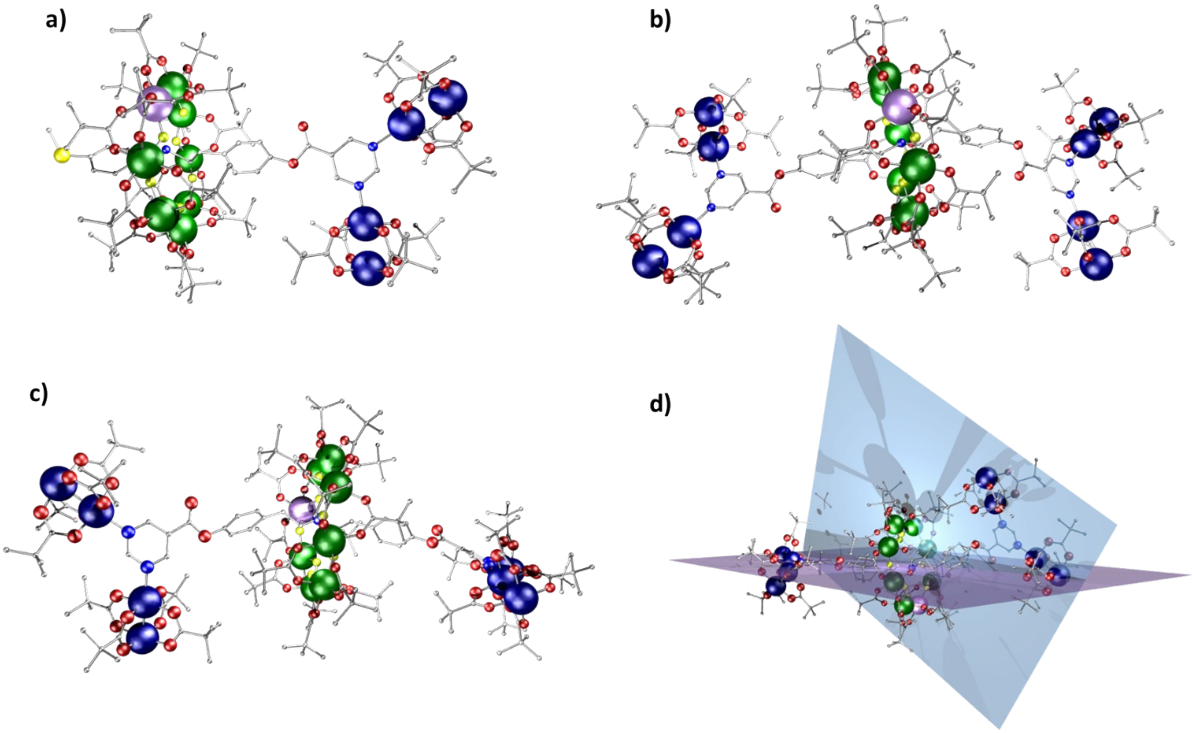

Figure S13. Figures showing rotaxanes bound to isolated Cu paddle wheel units, for a) **4**, b) **7** and c) **8**. Showing planes of the two pyrimidines for d) **8.** Atom colours: Cr; Green, Ni; Violet, Cu; Navy blue, O; Red, F; Yellow, C; Grey, N; Blue and S; Dull yellow. Hydrogens omitted for clarity

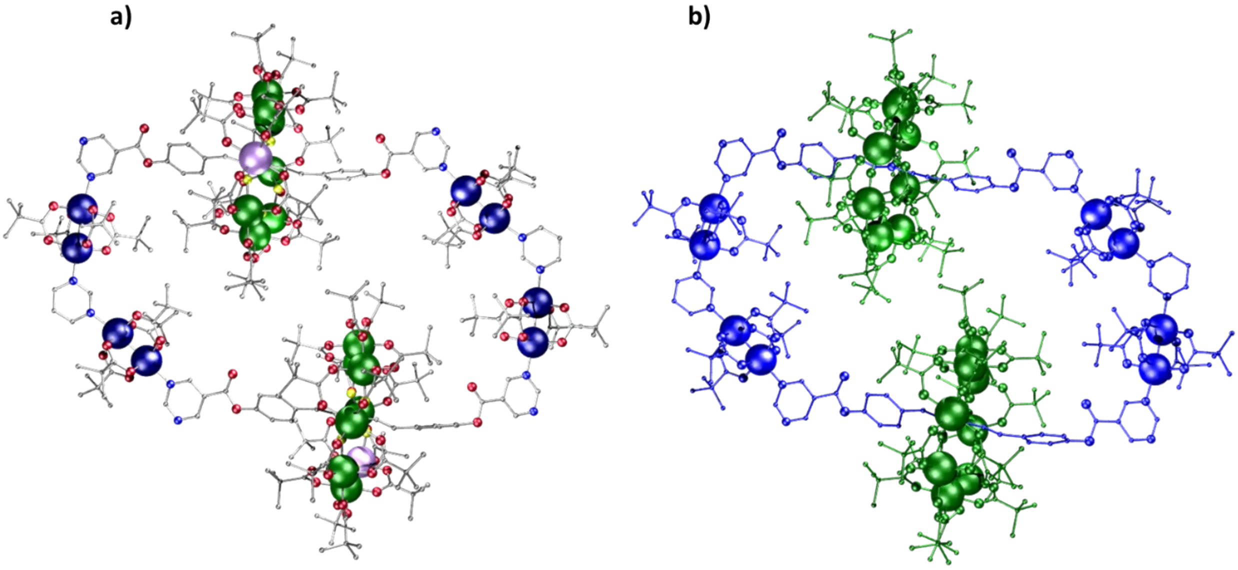

Figure S14. Figure showing the 6,3 net section for the 2D polymer **7**, a) atom colours: Cr; Green, Ni; Violet, Cu; Navy blue, O; Red, F; Yellow, C; Grey and N; Blue, and b) Framework depicted in blue, $\{Cr_7Ni\}$ rings in green. Hydrogens omitted for clarity.

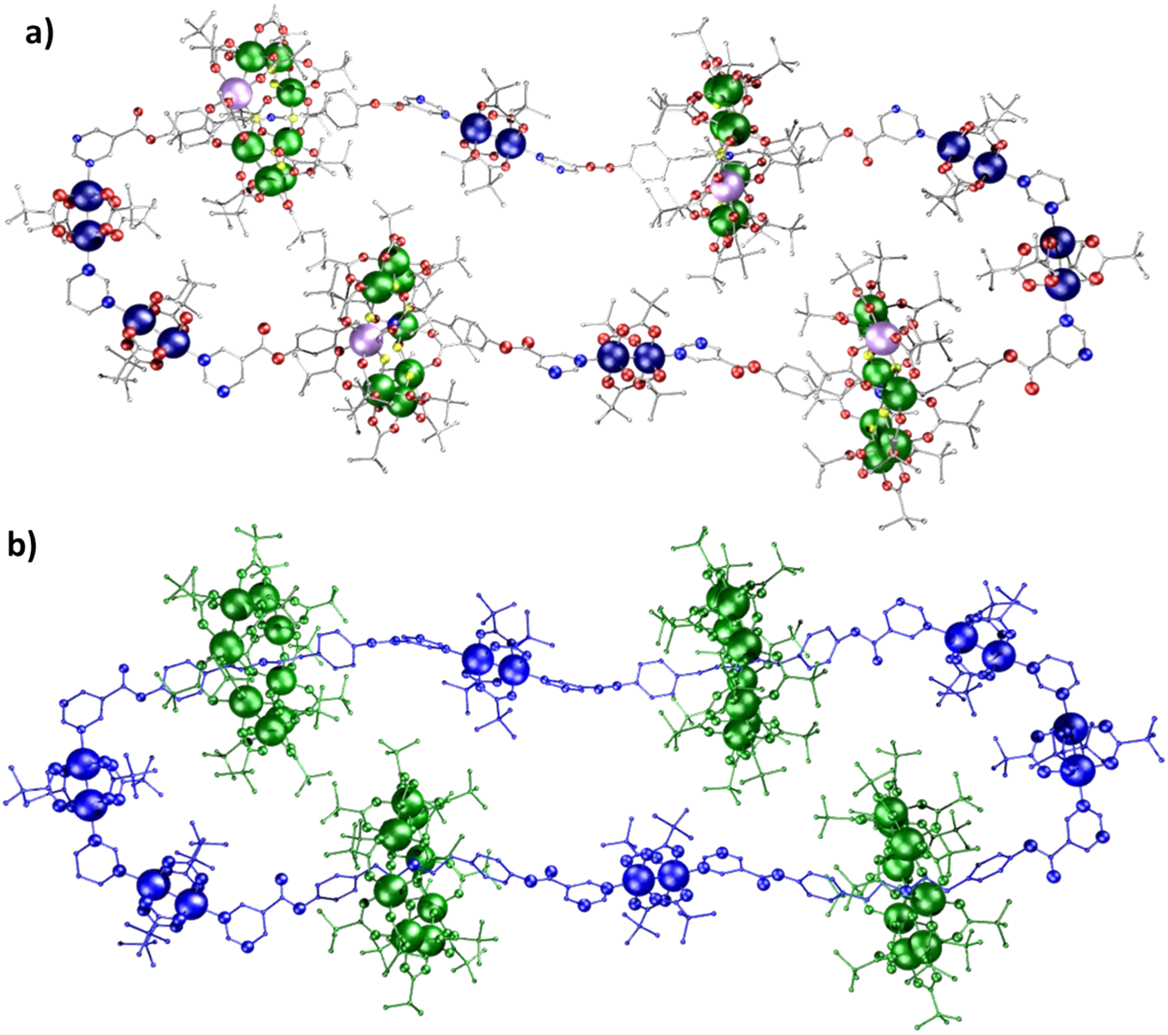

Figure S15. Figure showing the 10,3 net section for the 3D polymer **8**, a) atom colours: Cr; Green, Ni; Violet, Cu; Navy blue, O; Red, F; Yellow, C; Grey and N; Blue, and b) Framework depicted in blue, {Cr_7Ni} rings in green. Hydrogens omitted for clarity.

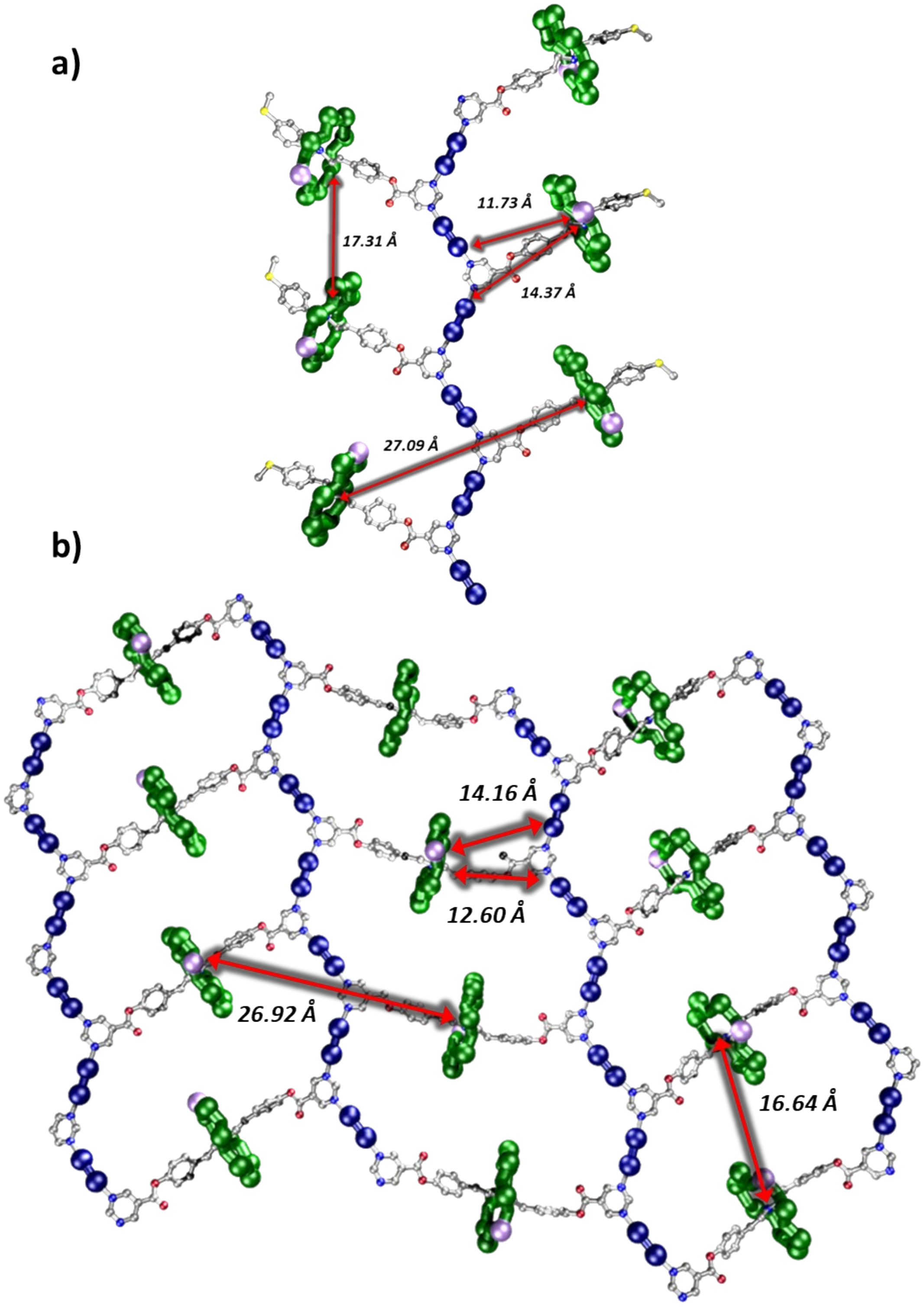

Figure S16. Section of the structures, showing intra; ring-ring (center-centre) distances, ring-copper (centre to nearest contact) and copper paddle wheel-copper paddle wheel (nearest contacts). For a) **4** and c) **7**.

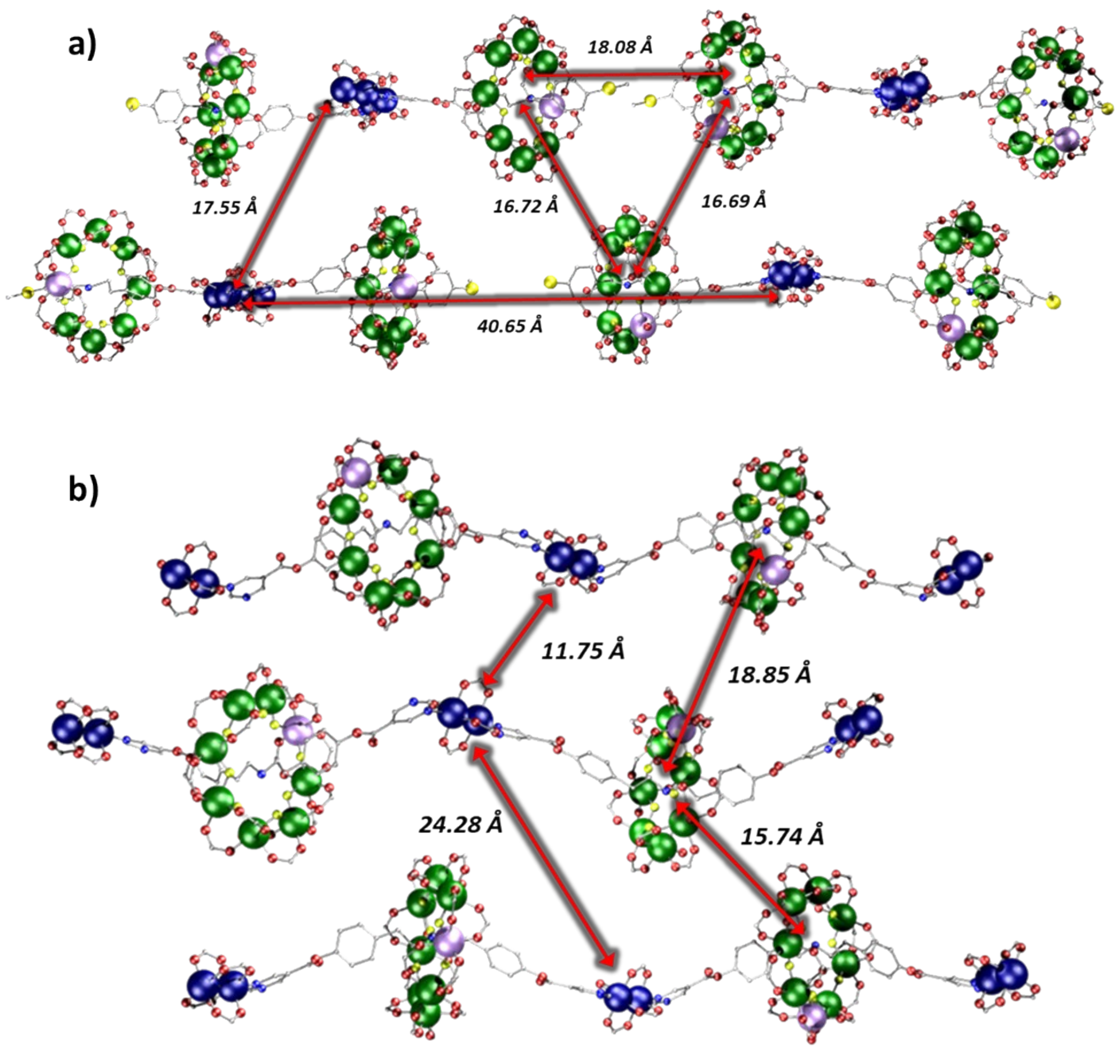

Figure S17. Figure showing the inter-layer contacts for a) **4** and b) **7**. Atom colours: Cr; Green, Ni; Violet, Cu; Navy blue, O; Red, F; Yellow, C; Grey, S; Dull yellow and N; Blue. Hydrogens omitted for clarity.

3) Electron Paramagnetic Resonance Measurements

Continuous wave Q-band (~34 GHz) EPR spectra were recorded with a Bruker EMXPlus spectrometer equipped with a Bruker ER5106QT flexline resonator. Cryogenic temperatures are achieved using a Bruker Stringer closed cycle helium cryocooler mated to an Oxford Instruments CF935 cryostat. Temperature holding and regulation are controlled using an Oxford Instruments MercuryITC. The continuous wave data were collected on polycrystalline powders at 5 K (unless otherwise stated). All continuous wave spectra were field corrected using a Bruker 'Strong Pitch' standard (g = 2.0028) and all powder samples were checked for any polycrystalline nature, by measuring multiple random rotations.

Spectral simulations were performed using the EasySpin 6.0.0 software[S4] with a spin Hamilton for **4**, **7** and **8** incorporating the individual *g*-matrices for the {Cr_7Ni} ring at 5K, and incorporating the individual *g*-matrices for the {Cr_7Ni} ring and Cu dimer, the Cu dimer hyperfine and zero field splitting interactions for the Cu dimer, at 150K and 297K, respectively:

$$\hat{H} = \mu_B \sum_i \hat{S}_i^{Cu} \cdot g^{Cu} \cdot B + \mu_B \sum_i \hat{S}_i^{Cr7Ni} \cdot g^{Cr7Ni} \cdot B + \sum_i \hat{S}_i^{Cu} \cdot A^{Cu} \cdot \hat{I}_i^{Cu}$$

$$\hat{H} = \mu_B \sum_i \hat{S}_i^{Cu} \cdot g^{Cu} \cdot B + \mu_B \sum_i \hat{S}_i^{Cr7Ni} \cdot g^{Cr7Ni} \cdot B + \sum_i \hat{S}_i^{Cu} \cdot A^{Cu} \cdot \hat{I}_i^{Cu} + \sum_i \hat{S} D \hat{S}$$

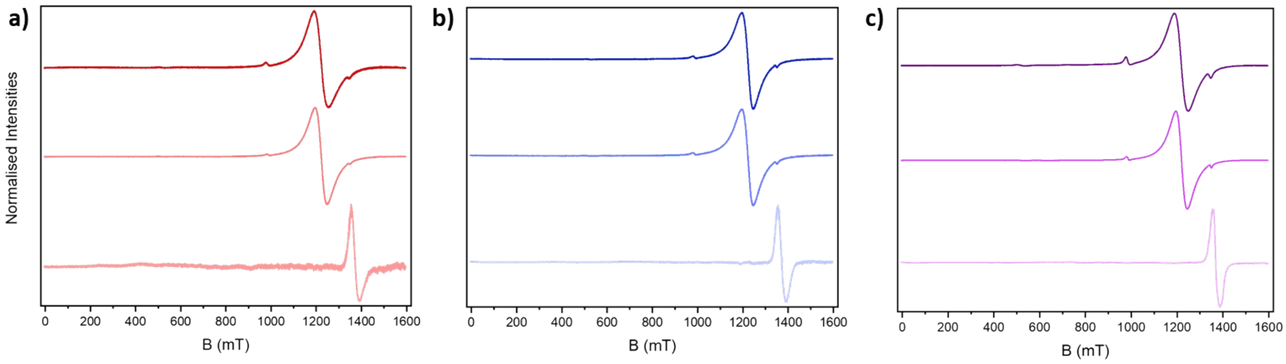

Figure 18. Continuous Wave EPR, Q-Band (c.a. 34 GHz), powder samples for **4**, a) **7**, b) and **8** c). Measured at temperatures of 297K, 150K and 5K for top, middle and bottom spectra, respectively.

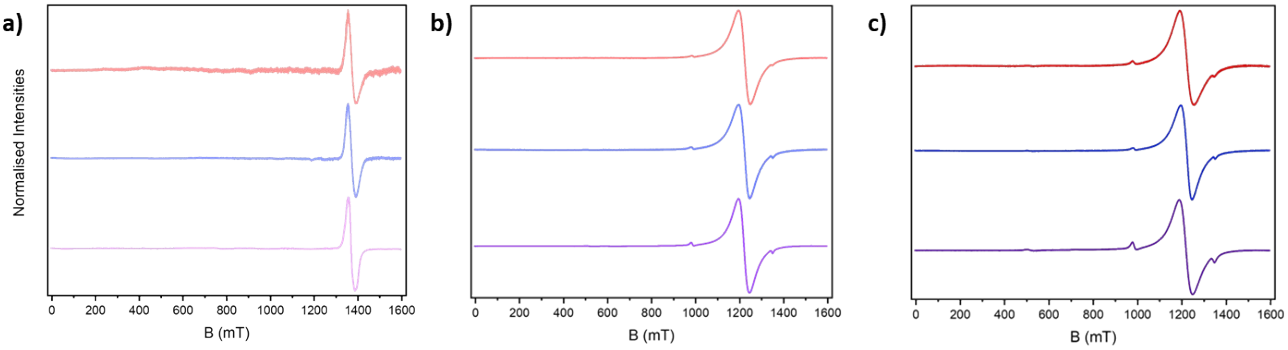

Figure 19. Continuous Wave EPR, Q-Band (c.a. 34 GHz), powder samples at a) 5K, b) 150K and c) 297K. Top, middle and bottom spectra for **4**, **7** and **8**, respectively.

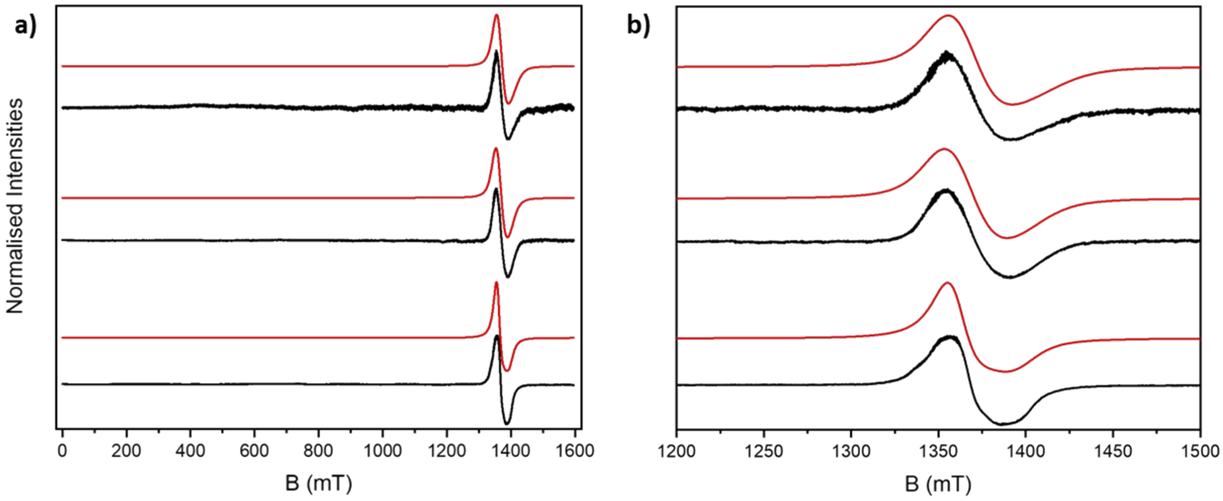

Figure S20. Continuous Wave EPR, Q-Band (c.a. 34 GHz), powder samples at 5K, a) full field width and b) 1200 – 1500 mT. Black line; experimental, red line; simulation. Top, middle and bottom spectra for **4**, **7** and **8**, respectively.

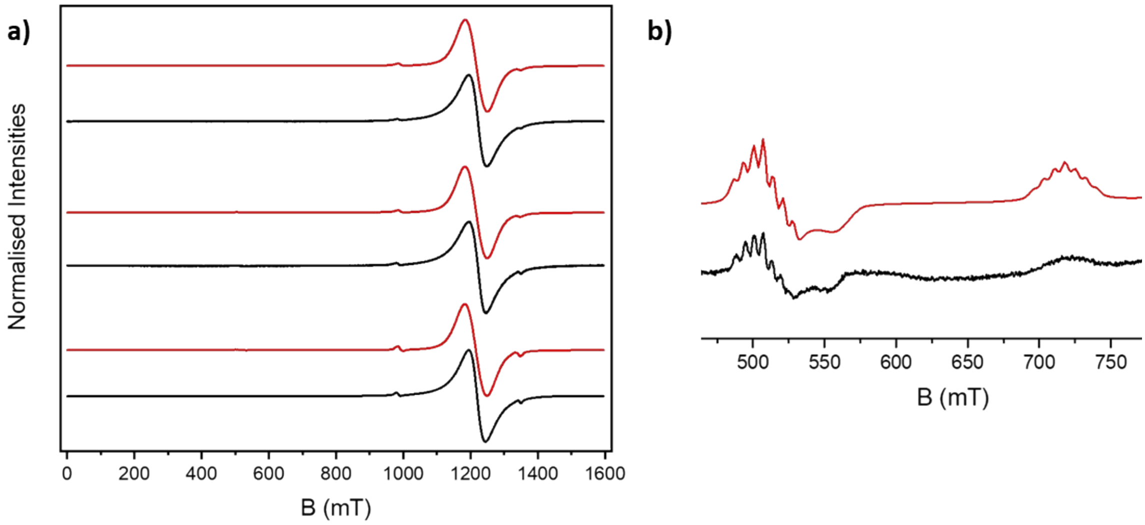

igure 21. Continuous Wave EPR, Q-Band (c.a. 34 GHz), powder samples at 150K, a) full field width. Top, middle and bottom spectra for **4**, **7** and **8**, respectively. b) 470 – 770 mT for **8**. Black line; experimental, red line; simulation.

Table S2.

Simulation Parameters (5K)	4	7	8
$g^{\{Cr_7Ni\}}_{xyz}$	1.780, 1.780, 1.735	1.785, 1.785, 1.745	1.788, 1.788, 1.745
$gStrain^{\{Cr_7Ni\}}_{xyz}$ (%)	3, 3, 5	3, 3, 4	1, 1, 2
Linewidth (mT)	25	25	25

Table S3.

Simulation Parameters (150K)	4	7	8
$g^{\{Cr_7Ni\}}_{xyz}$	2.00, 2.00, 2.00	2.00, 2.00, 2.00	2.00, 2.00, 2.00
$gStrain^{\{Cr_7Ni\}}_{xyz}$ (%)	10, 10, 10	10, 10, 10	10, 10, 10
$g^{Cu\ dimer}_{xyz}$	2.06, 2.06, 2.37	2.06, 2.06, 2.37	2.06, 2.06, 2.37
$^{Cu}A_{xyz}$ (MHz)	N/A	N/A	100, 100, 240
D^{Cu} (MHz)	-10,200	-10,200	-10,200
Linewidth: $\{Cr_7Ni\}$ / Cu Dimer (mT)	25 / 15	25 / 15	25 / 4

*Simulation parameters given for 150K are the same for 297K

4) References

S1. G. A. Timco, S. Carretta, F. Troiani, F. Tuna, R. J. Pritchard, C. A. Muryn, E. J. L. McInnes, A. Ghirri, A. Candini, P. Santini, G. Amoretti, M. Affronte, R. E. P. Winpenny, Engineering the coupling between molecular spin qubits by coordination chemistry, *Nature Nanotech*. , 2009, **4**, 173–178.

S2. (a) G. M. Sheldrick, SADABS, empirical correction for absorption anisotropy. (b) L. Krause, R. Herbst-Irmer, G. M. Sheldrick, D. Stalke, An empirical correction for absorption anisotropy, *J. Appl. Cryst*., 2015, **48**. (c) R. H. Blessing, An empirical correction for absorption anisotropy, *Acta Crystallogra*., 1995, **A51**, 33-38.

S3. (a) G. M. Sheldrick, Crystal structure refinement with SHELXL, *Acta Crystallogr*., 2015, **C71**, 3-8; (b) O. V. Dolomanov, L. J. Bourhis, R. J. Gildea, J. A. K. Howard, H. Puschmann, OLEX2: a complete structure solution, refinement and analysis program, *J. Appl. Cryst*., 2009, **42,** 339-341.

S4. S. Stoll, A. Schweiger, Easyspin, a comprehensive software package for spectral simulation and analysis in EPR, *J. Magn. Reson*., 2006, **178**, 42-55.
